# Highly sensitive magnetic particle imaging of abdominal aortic aneurysm NETosis with anti-Ly6G iron oxide nanoparticles

**DOI:** 10.1038/s41420-024-02156-3

**Published:** 2024-09-05

**Authors:** Heng Wang, Ruijing Zhang, Xiaohua Jia, Siqi Gao, Tingting Gao, Keyi Fan, Yaling Li, Shule Wang, Maolin Qiao, Sheng Yan, Hui Hui, Honglin Dong

**Affiliations:** 1 Department of Vascular Surgery, The Second Hospital of Shanxi Medical University, Taiyuan, 030001 China; 2 Department of Nephrology, The Second Hospital of Shanxi Medical University, Taiyuan, 030001 China; 3 Key Laboratory of Molecular Imaging of Chinese Academy of Sciences, Institute of Automation, Chinese Academy of Sciences, Beijing, 100190 China; 4 Department of Ultrasound, Shuozhou Grand Hospital of Shanxi Medical University, Shuozhou, 036000 China; 5 National Key Laboratory of Kidney Diseases, Beijing, 100853 China

**Keywords:** Aneurysm, Cell death

## Abstract

Abdominal aortic aneurysms (AAA) are a significant health concern in developed countries due to their considerable mortality rate. The crucial factor of the progression of AAA is the release of neutrophils and neutrophil extracellular traps (NETs). Magnetic particle imaging (MPI) is a new imaging technique that offers the capability to detect superparamagnetic iron oxide nanoparticles (SPION) with exceptional sensitivity. We aimed to investigate the functional imaging of MPI for the detection and monitoring of neutrophil infiltration within AAA. A novel multimodal imaging agent targeting neutrophils, PEG-Fe_3_O_4_-Ly6G–Cy7 nanoparticles (Ly6G NPs), were designed by coupling Fe_3_O_4_ nanoparticles with Ly6G antibodies and Cy7. The targeting and sensitivity of Ly6G NPs were assessed using MPI and fluorescence imaging (FLI) in the AAA mouse model. After the inhibition of NETosis, the degree of neutrophil infiltration and AAA severity were assessed using MPI with Ly6G NPs. Ly6G NPs accurately localized and quantitatively analyzed AAA lesion sites in mice using MPI/FLI/CT. Compared to the control group, elevated MPI and FLI signal intensities were detected at the abdominal aortic lesion site, and neutrophil infiltration and NETs accumulation were detected by histological analysis in the AAA models. After the inhibition of NETs accumulation in vivo, pathological damage in the abdominal aorta was significantly reduced, along with a decrease in the accumulation of Ly6G NPs and MPI signals. This multimodal MPI strategy revealed that nanoparticles targeting Ly6G can be used to detect neutrophil infiltration within AAA and monitor AAA severity.

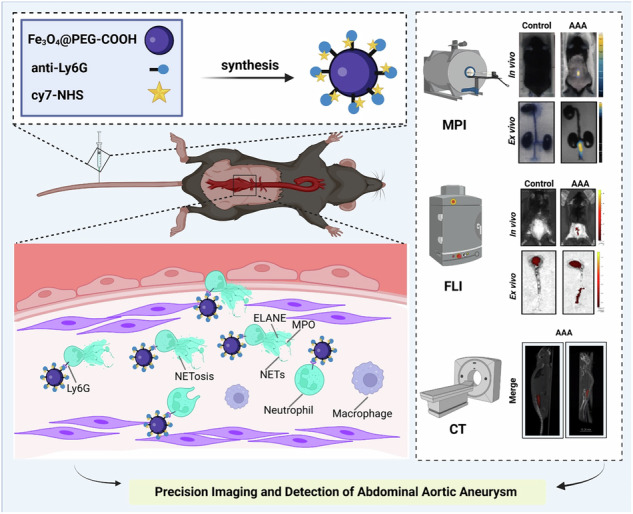

## Introduction

An aneurysm represents a persistent and irreversible condition characterized by the dilation of arteries. Of these, aneurysms that occur in the segment of the abdominal aorta located below the renal arteries are known as abdominal aortic aneurysms (AAA), which is the most common site. In 1991, the Society for Vascular Surgery and the International Society for Cardiovascular Surgery Ad Hoc Committee on Standards in Reporting defined AAA when the infrarenal aorta is dilated to 1.5 times its original diameter [1]. Although it is usually asymptomatic, its major complication, aortic dissection, has a mortality rate of 65–85%, with 150,000–200,000 deaths from aortic dissection each year worldwide [2, 3]. Because of its highly insidious nature, imaging is critical for early screening and dynamic monitoring of AAA [4].

Current imaging for AAA focuses on vascular morphology. Regular abdominal ultrasound imaging is commonly considered the primary method for both detecting and tracking AAA in individuals without symptoms [5]. Computed tomography (CT) is an important reference standard for detecting concurrent aneurysmal disease [6]. New studies have emerged that use functional imaging to determine the probability of AAA expansion and rupture. The accumulation of the glucose analog 18F-FDG, one of the most utilized radiotracers in positron emission tomography (PET) and PET-CT imaging, can identify sites of increased glycolysis, such as sites of inflammation in AAA [7]. However, it is challenging to perform AAA imaging because of the background noise and nonspecific uptake of 18F-FDG [8]. In addition, ultrasmall iron oxide (USPIO)-based magnetic resonance imaging (MRI) is a novel approach to identifying cellular inflammation within the aortic wall of individuals with AAA and predicting the aneurysm growth rate and clinical prognosis [9]. However, MRI tends to rely on the surrounding tissue, which is difficult to distinguish from air, artifacts, and pathological tissue. Therefore, it is crucial to find a functional imaging modality that is specific, sensitive, and safe [10].

Magnetic particle imaging (MPI) represents a novel tomographic technique that utilizes superparamagnetic iron oxide nanoparticles (SPION) as imaging agents for highly sensitive detection and quantitative analysis [11, 12]. The concept of MPI was first proposed in 2005, and in recent years it has been employed mostly for preclinical study, not extending significantly into clinical investigations [13]. MPI has the advantages of high sensitivity, zero tissue-depth signal attenuation, absence of ionizing radiation, and quantifiable analysis [14]. In addition, in combination with vascular anatomy imaging using ultrasound, CT, and MRI, MPI can be used as an imaging technique for the quantitative assessment of vascular function. Using ferucarbotran as an imaging agent, Mangarova et al. first reported the application of MPI in the visualization of vascular inflammation in isolated AAA in mice [15]. Importantly, Tong et al. specifically recognized atherosclerosis-prone plaques in the mouse abdominal aorta by constructing iron oxide nanoparticles targeting myeloperoxidase (MPO) [16]. In a follow-up study, MPI detected intraplaque hemorrhage in vulnerable plaques of the carotid arteries in mice without the aid of an exogenous probe and was validated in human carotid endarterectomy postoperative specimens [17]. These studies confirmed the feasibility of AAA for functional imaging of MPI and the importance of identifying specific targeting probes for in vivo experimental validation.

The main pathophysiological features of AAA are protein degradation in the extracellular matrix (ECM), vascular smooth muscle cell (VSMC) death, and infiltration of immune cells [3, 4, 18]. Nearly three-quarters of AAA show the development of intraluminal thrombus (ILT), which includes a substantial quantity of neutrophils, macrophages, and other inflammatory cells [19, 20]. Neutrophils infiltrate the aorta and release a variety of protein hydrolases that lead to VSMC phenotypic transformation or death, and promote ECM degradation [21–23]. Thus, the role of neutrophils in AAA has become very crucial. When faced with pathogens, neutrophils undergo NETosis and release neutrophil extracellular traps (NETs), a meshwork of DNA, histones, and granular proteins. This process differs from other modes of cell death [24]. Neutrophil extracellular traps (NETs) are a host defense mechanism that ensnares and eliminates pathogens using adhesive NETs containing killer proteins and is also significant in aseptic inflammation [25]. It has been found that NETs induce iron death in VSMC through inhibition of the PI3K/AKT pathway and promote synthetic and pro-inflammatory VSMC formation through the Hippo-YAP pathway, thereby attenuating vessel wall elasticity [26, 27]. In addition, NETs are involved in intraluminal thrombosis, promote the infiltration of inflammatory cells (macrophages and plasma cell-like dendritic cells), and degrade the ECM [28, 29]. Thus, neutrophils are potentially valuable functional imaging biomarkers for assessing AAA.

**Fig. 1:**
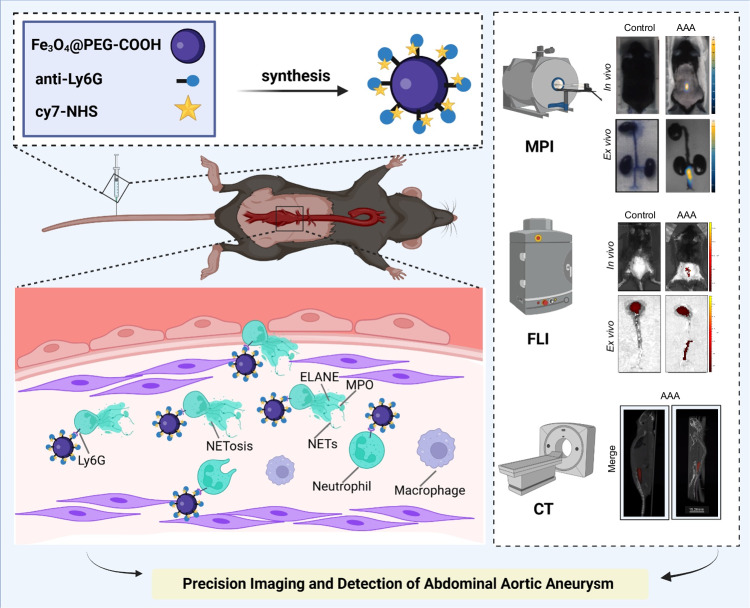
Schematic illustration of MPI/FLI/CT imaging of infiltrating neutrophils to identify AAA. Targeted nanoprobes PEG–Fe_3_O_4_–Ly6G–Cy7 (Ly6G NPs) were synthesized by coupling Ly6G antibody with Fe_3_O_4_@PEG–COOH and Cy7–NHS. Ly6G NPs were injected into AAA mice via the tail vein and specifically bound to neutrophils infiltrated in diseased vessels. AAA was functionally imaged in vivo by MPI/FLI/CT imaging. Created with *BioRender.com*.

In our research, we created a novel multimodal AAA imaging platform to identify AAA ductal wall inflammation by detecting the neutrophil surface marker (Ly6G) (Fig. 1). The novel probe, PEG–Fe_3_O_4_–Ly6G–Cy7 (Ly6G NPs), consists of an antibody anti-Ly6G that actively targets Ly6G, iron tetraoxide nanoparticles (Fe_3_O_4_@PEG–COOH), and a fluorescent moiety (Cy7–NHS). The probes that noninvasively labeled neutrophils in mice were significantly enriched in AAA lesions and quantitatively responded to AAA severity.

## Results

### Neutrophil infiltration and NETs release aggravate AAA

**Fig. 2:**
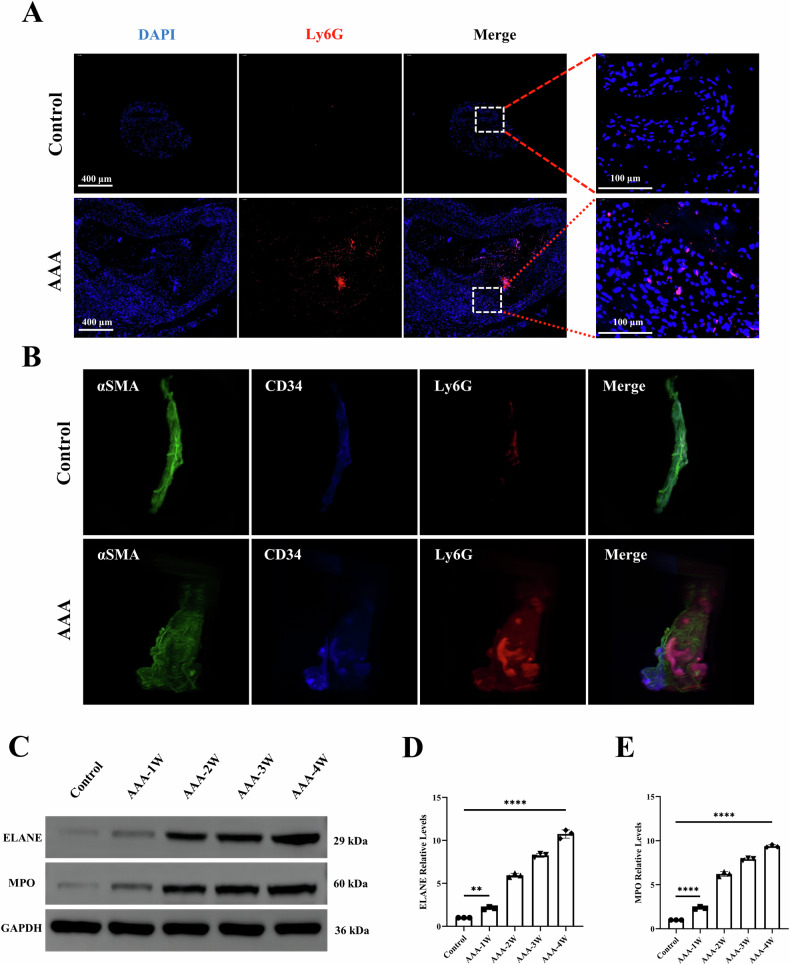
Neutrophil infiltration and NETs release within AAA lesions. **A** IF staining for neutrophil marker (Ly6G) was performed on tissue sections of the mouse abdominal aorta. **B** Hyalinization and IF labeling of the whole abdominal aorta and 3D tissue imaging were acquired with light-sheet microscopy. **C**–**E** Quantification of NETs markers (ELANE and MPO) in mouse abdominal aorta by western blotting (*n* = 3). **P < 0.01, ****P < 0.0001, indicating statistical significance between the two groups.

IF staining Ly6G of the diseased segment of AAA mouse vessels revealed intraluminal thrombi with neutrophil aggregates and intramural neutrophil infiltration (Fig. 2A). We then did tissue hyalinization of the whole segment of diseased aorta, and after IF labeling with antibodies against CD34, αSMA, and Ly6G, 3D imaging was performed using light-sheet microscopy (Fig. 2B). The results showed neutrophil infiltration and accumulation in the AAA lesions. Meanwhile, it was performed IF co-localization of neutrophils and NETs (CitH3 and MPO), and the results showed that NETs were produced within the AAA vessel walls (Fig. S1).

Next, we examined NET expression at different stages of AAA. AAA modeling was performed using the elastase incubation method, and dilation of the infrarenal abdominal aorta was clearly observed over time (Fig. S2A). As detected by small-animal ultrasound, the internal diameter of the abdominal aorta increased one week after modeling, and dilatation became more pronounced in the fourth week (Fig. S2B, C). Further pathological staining of the abdominal aorta revealed elastin degradation, disorganization of elastic fiber breaks, smooth muscle cell proliferation, and increased collagen fiber secretion (Fig. S2D). In addition, inflammatory markers IL-6 and TNF-α were measured in the serum of mice, and their expression was found to increase as AAA progressed (Fig. S2E, F). This suggests that the pathological damage of AAA increases with prolonged modeling time.

WB was performed on abdominal aortic tissues at different time points. As AAA progressed, the protein expression levels of ELANE and MPO, the main components of NETs, gradually increased (Fig. 2C–E). ELISA detection of MPO in the mouse serum showed the same trend (Fig. S2G). This suggests that NETs are involved in AAA progression and are positively correlated with severity.

### Physical characterization of Ly6G NPs

**Fig. 3:**
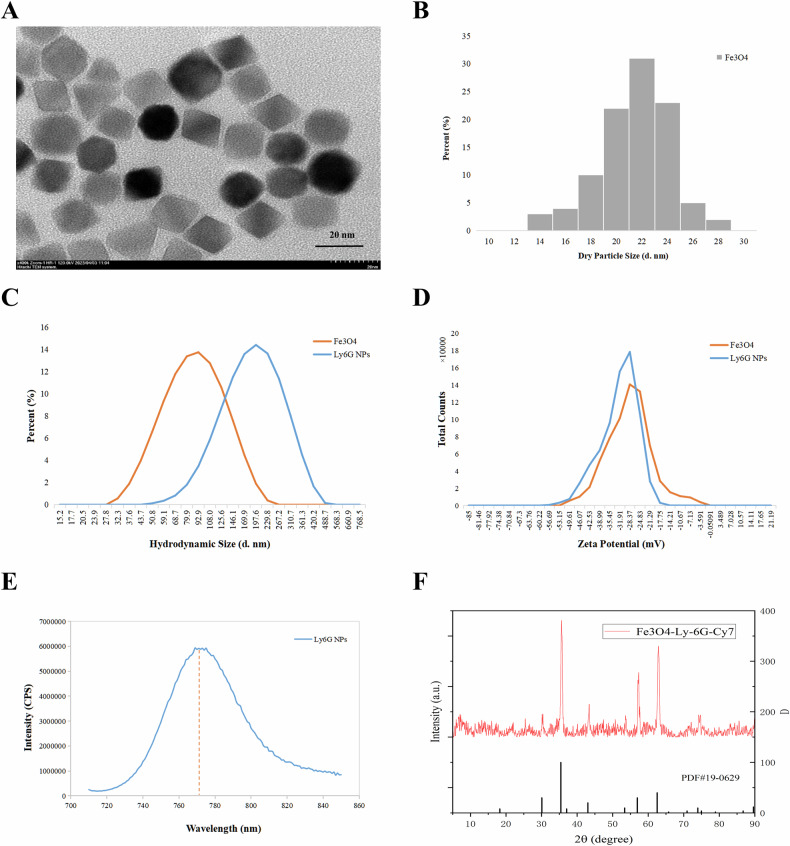
Physical characterization of Ly6G NPs. **A**, **B** TEM images and size distribution of Ly6G NPs. **C**, **D** Hydrated particle size and zeta potential of Ly6G NPs. **E** Emission spectra of Ly6G NPs at 700 nm excitation light. **F** Powder X-ray diffraction (XRD) patterns of Ly6G NPs.

We chose 20 nm PEGylated magnetic nanoparticles coupled with the antibody Ly6G and then Cy7 fluorescence modification to form PEG–Fe_3_O_4_–Ly6G–Cy7 nanoparticles (Ly6G NPs). The TEM results showed that most of the particle sizes of the Ly6G NPs in the dry state were in the range of 20–24 nm, with good particle dispersion (Fig. 3A, B). The hydrated particle size results showed that the Intensity of the original Fe_3_O_4_ nanoparticles was 100 nm and the Zeta potential was −27.66 mV; after the antibody coupling and modification of the fluorescent Cy7, the Intensity was increased to 197.6 nm, and the Zeta potential was decreased to −31.24 mV (Fig. 3C, D; Tables S1 and 2). The probe-antibody coupling rate was 93.75%, based on antibody detection of the probe supernatant (Table S3). Excitation at a set wavelength of 700 nm and scanning of the emission spectrum at 710–850 nm, there was a maximum emission light at 775 nm, which was consistent with the Cy7 fluorescence spectrum (Fig. 3E). In addition, in the XRD pattern, the size and position of the main diffraction peaks of the Ly6G NPs coincided with the characteristic diffraction peaks of standard Fe_3_O_4_ (Fig. 3F). Diffraction peaks appeared at diffraction angles of approximately 30°, 35°, 43°, 53°, 57°, and 62°, which correspond to the diffraction of Fe_3_O_4_ 220, 311, 400, 422, 511, and 440 in the crystallographic directions, respectively.

The above data illustrate that the PEG–Fe_3_O_4_–Ly6G–Cy7 composite nanoparticles (Ly6G NPs) were prepared with a homogeneous particle dispersion, overall negative charge, and 93.75% antibody coupling rate.

### In vitro stability, biocompatibility, and in vivo specificity of Ly6G NPs

**Fig. 4:**
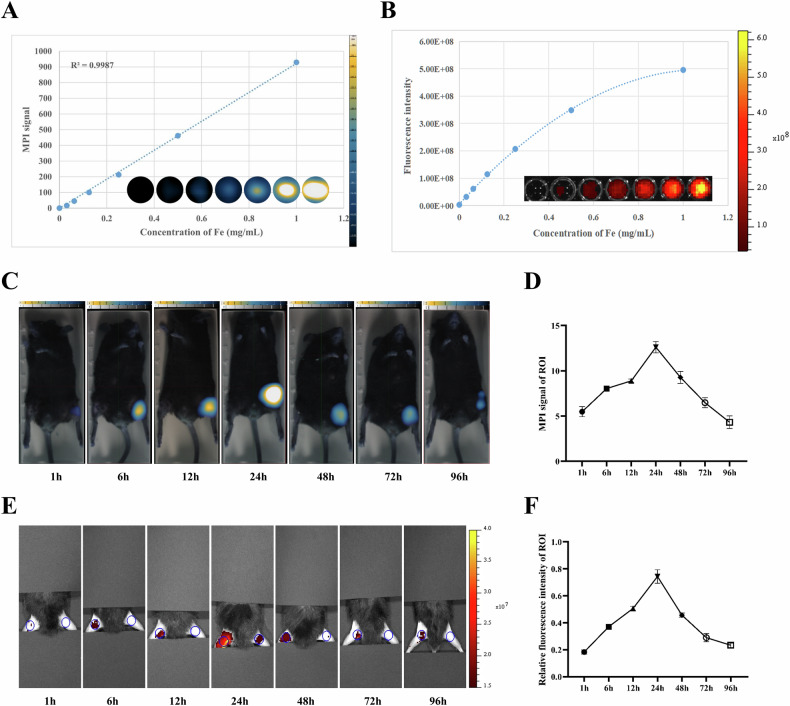
Ex vivo stability and in vivo specificity of Ly6G NPs. **A**, **B** MPI and FLI signals and corresponding curves for different concentrations of Ly6G NPs in test tubes. **C**–**F** MPI and FLI imaging were performed after mice were injected intramuscularly in the left leg with a matrix gel containing Ly6G proteins and lipopolysaccharides, followed by injection of Ly6G NPs in the tail vein (*n* = 3).

To investigate the stability of Ly6G NPs in vitro, we detected MPI signals at iron concentrations of 0, 0.03125, 0.0625, 0.125, 0.25, 0.5, and 1 mg/mL and observed a stable linear correlation, with *R*^2^ = 0.9987 (Fig. 4A). As shown in Fig. 4B, the fluorescence signals were gradually enhanced and finally stabilized with the increase of Ly6G NPs concentration. The Ly6G NPs were injected via the tail vein into healthy mice at a dose of 7.5 mg Fe/kg. The MPI scanning results showed that the peak signal value was reached at 24 h, after which it was gradually metabolized and still had a high MPI signal value at 15 days (Fig. S3A, B). After incubating mice peripheral blood neutrophils with different concentrations of Ly6G NPs, cell activity was detected at 6 h. The CCK8 assay results showed that the probe did not have a significant cytotoxic effect (Fig. S3C).

Normal mice injected with Ly6G NPs for 24 h were necropsied, and the organs were imaged ex vivo using MPI, which showed high signals in the liver and spleen (Fig. S3D). Mice with their livers and spleens removed were scanned using MPI, which showed essentially no signal (Fig. S3E). In addition, ex vivo organ imaging using FLI showed strong signaling in the small intestine, stomach, liver, and spleen (Fig. S3F). Various organs were sampled from mice at 24 h and 15 days after injection, and HE staining showed that Ly6G NPs did not cause acute or chronic damage to the various organs of mice, and the probes were mainly enriched in the liver and spleen (Fig. S4). Serum from mice at 24 h after injection were obtained and tested for renal and hepatic function indicators, confirming the biocompatibility of Ly6G NPs (Fig. S5).

To assess the targeting specificity of Ly6G NPs in mice, Ly6G proteins and lipopolysaccharides were encapsulated in matrix gels and injected into the left leg of mice. The injection of Ly6G NPs via the tail vein after 12 h resulted in significantly higher MPI and FLI signals in the left legs of mice than in the healthy right legs of mice over 1–96 h. The MPI and FLI signals reached their maximum at 24 h after the probe injection (Fig. 4C–F).

### Targeted identification of AAA in mice by MPI/FLI/CT imaging based on Ly6G NPs

**Fig. 5:**
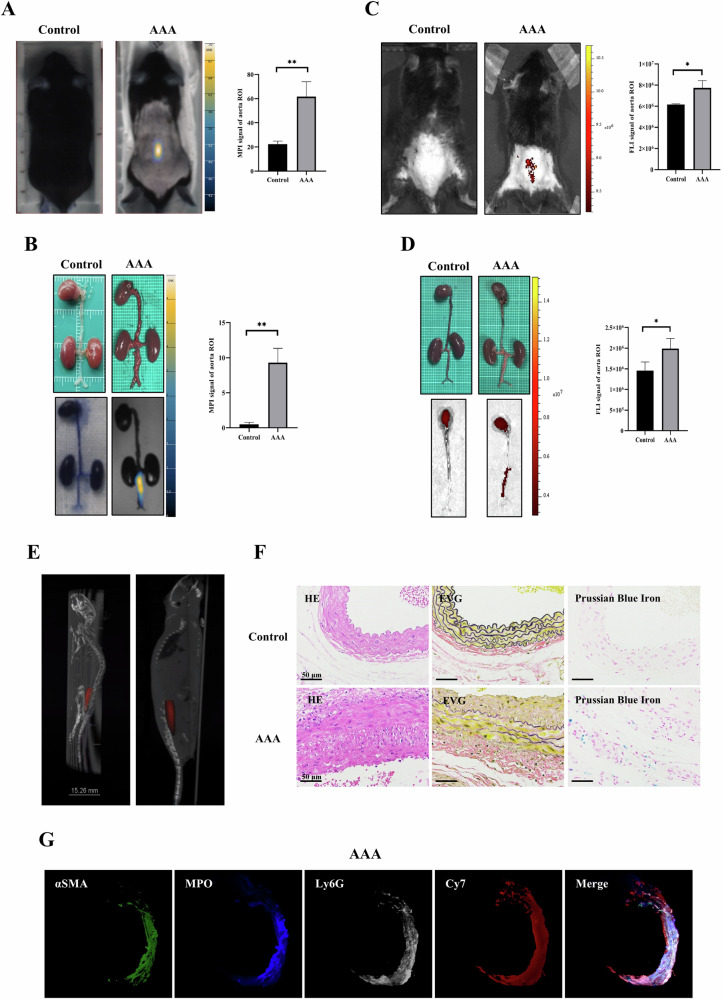
MPI/FLI/CT imaging of AAA lesions in mice by intravenous injection of Ly6G NPs. **A** MPI in vivo imaging of normal mice and AAA was performed 24 h after injection of Ly6G NPs (*n* = 3). **B** Mice were necropsied, and the aorta was isolated intact for ex vivo MPI scanning imaging (*n* = 3). **C** FLI in vivo imaging was performed on normal mice and AAA 24 h after injection of Ly6G NPs (*n* = 3). **D** Mice were necropsied, and aortas were isolated intact for ex vivo FLI scanning imaging (*n* = 3). **E** The fused images of MPI 3D data with CT data of AAA mice. **F** Tissue sections of abdominal aorta from normal and AAA mice were stained with H&E, EVG, and Prussian blue, respectively. **G** AAA mice injected with Ly6G NPs 24 h after the injection were put to death, and the diseased vessels were hyalinized and labeled by IF, and then 3D tissue imaging was acquired by light-sheet microscopy.

AAA model mice underwent MPI/FLI/CT imaging four weeks after modeling. MPI images were acquired 24 h after the injection of Ly6G NPs via the tail vein. On MPI scans, the anatomical location of the abdominal aorta in the AAA model mice presented higher MPI signals (Fig. 5A). After anesthetizing and sacrificing the mice, ex vivo MPI imaging of the stripped heart, kidney, and aorta detected higher signal values only in the AAA lesion (Fig. 5B). FLI images were acquired 12 h after the injection of Ly6G NPs via the tail vein. Similarly, we observed stronger Cy7 fluorescence signals in the backs of AAA mice (Fig. 5C). The isolated abdominal aorta was imaged again, and the dilated region showed a strong fluorescent signal (Fig. 5D). The 3D data of the AAA mouse MPI were then fused with the CT data, and it was found that the MPI signals were located at the anatomical site of the abdominal aorta in the corresponding infrarenal segment (Fig. 5E; Video 1). In both In vivo and Ex vivo imaging, FLI and MPI signal values increased as AAA progressed (Fig. S6). Besides, we constructed BSA NPs Ly6G as a negative control and injected it into AAA-4w mice via tail vein, and compared it with the MPI/FLI signals of Ly6G NPs at 24 h after injection, which showed that the Ly6G NPs had a clear targeting specificity toward AAA (Fig. S7). In addition, we performed pathological staining of the abdominal aortic tissue, and Prussian blue staining further revealed the presence of Ly6G NPs mainly in the lesion area (Fig. 5F). The 3D fluorescence reconstruction showed that the Ly6G^+^ and Cy7^+^ regions overlapped in AAA, indicating that the Ly6G NPs targeted the recognition of the neutrophil marker Ly6G (Fig. 5G).

### Improving the pathological progression of AAA by inhibiting the occurrence of NETosis

Gross specimen analysis revealed that abdominal aortic dilatation was significantly reduced in mice after administration of exogenous NETs inhibitors and scavengers (Fig. S8A). Small-animal ultrasound statistics further demonstrated that the lumen diameter showed a significant decrease in the treatment group as opposed to the AAA group (Fig. S8B–D). Pathological staining showed thinning of the vascular wall musculature and reduced proliferation of the smooth muscle layer in drug-treated mice compared with the model group (Fig. S8C). Serum ELISA showed that the level of inflammation in drug-treated mice was significantly reduced (Fig. S8E, F).

We then assayed the levels of NETs in mice. Serum ELISA demonstrated a marked reduction in MPO expression in the peripheral blood in the administered mice (Fig. S8G). In addition, the WB assay was performed on mouse aortic tissues, and the expression of ELANE and MPO, components of NETs, was significantly reduced in the administered group compared to that in the AAA group (Fig. S8H–J). This suggests that both Cl-Amidine and DNase I successfully reduced NET accumulation in the peripheral circulation and diseased tissues of AAA mice.

### MPI/FLI imaging to monitor neutrophil infiltration within aortic aneurysms

**Fig. 6:**
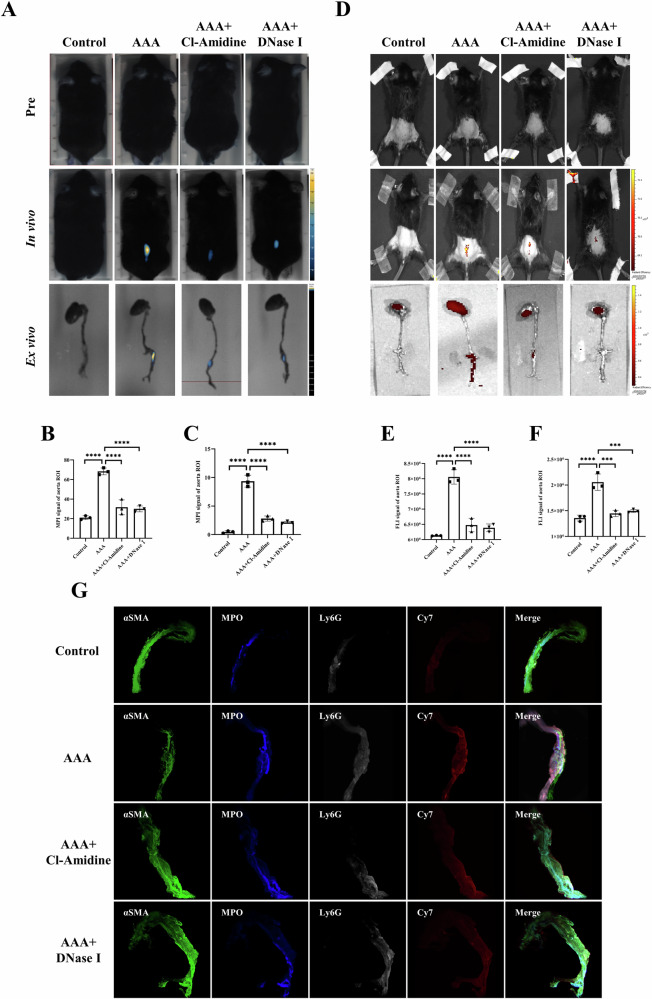
MPI/FLI imaging of different groups of mice after intravenous injection of Ly6G NPs. **A**–**C** In vivo and ex vivo MPI imaging and quantitative analysis of mice before and 24 h after injection of Ly6G NPs (*n* = 3). **D**–**F** In vivo and ex vivo FLI imaging and quantitative analysis of mice before and 24 h after injection of Ly6G NPs (*n* = 3). **G** 3D tissue IF imaging of abdominal aorta in different groups of mice. ***P < 0.001, ****P < 0.0001, indicating statistical significance between the two groups.

Eight-week-old male C57BL/6 J mice were specifically inhibited from NETs expression in vivo by the administration of the PADI4 inhibitor Cl-Amidine or the DNA degrader DNase I after AAA modeling. The abdominal aorta was examined three weeks after continuous intraperitoneal injection. Mice in the model and drug-treated groups were imaged 24 h after the tail vein injection of Ly6G NPs. MPI in vivo imaging was performed in mice. The MPI signal values were significantly elevated in the AAA group, whereas both treatment groups showed significantly reduced signal values in the AAA group (Fig. 6A, B). Ex vivo MPI imaging of isolated abdominal aortas after anesthetizing and sacrificing the mice demonstrated a significant decrease in signal values in the treatment group compared to the AAA group (Fig. 6A, C). In addition, we performed in vivo and ex vivo FLI imaging in mice. As with MPI imaging, the Cy7 fluorescence intensity was elevated in the AAA group than in the normal group, whereas the drug administration group showed significantly lower fluorescence signals (Fig. 6D–F). Meanwhile, in the 3D fluorescence reconstruction map Fig. 6G, Cl-Amidine and DNase I reduced the NETs component MPO^+^ region and decreased neutrophil infiltration and probe accumulation in the AAA (Video 2).

**Fig. 7:**
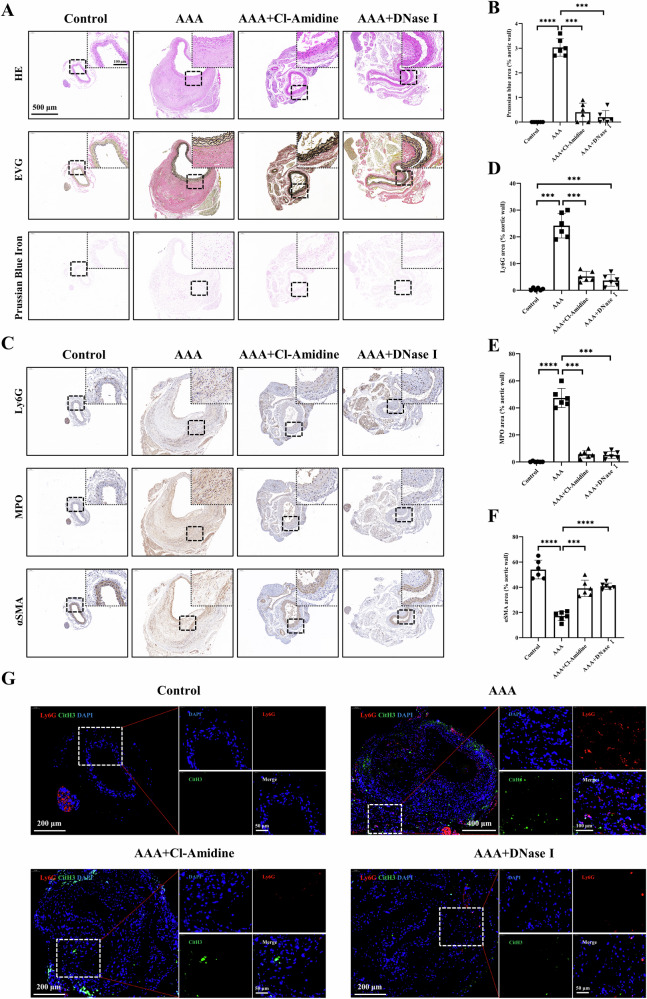
Histologic analysis of abdominal aorta sections from different groups of mice. **A** H&E, EVG, and Prussian blue staining of abdominal aorta sections from different groups of mice. The black dashed boxes are the corresponding magnified images. **B** Semi-quantitative analysis of Prussian blue-positive areas within the vessel wall (*n* = 6). **C** IHC staining of neutrophils (Ly6G), NETs (MPO), and vascular smooth muscle cells (αSMA) was performed on abdominal aorta sections from different groups of mice. **D**–**F** Semi-quantitative analysis of Ly6G-, MPO-, and αSMA-positive areas within the vessel wall, respectively (*n* = 6). **G** IF co-localization staining of neutrophils (Ly6G) and NETs (CitH3) on abdominal aorta sections from different groups of mice. ***P < 0.001, ****P < 0.0001, indicating statistical significance between the two groups.

Figure 7A shows the results of H&E, EVG, and Prussian blue staining of mouse aortas. Visible blue-stained particles can be seen in AAA, indicating the infiltration and accumulation of Ly6G NPs, while there are almost no blue-stained particles in the aorta of the treatment group (Fig. 7A, B). Figure 7C shows IHC staining results of Ly6G, MPO, and α-SMA in the abdominal aorta. The neutrophil marker Ly6G infiltrated the AAA lesion and was barely expressed in the control and treatment groups (Fig. 7C, D), similar to the Prussian blue staining. This suggests that Ly6G NPs preferentially accumulated in Ly6G-positive areas in the AAA group. MPO, a marker for NETs, showed a similar expression trend as Ly6G in all groups (Fig. 7C, E). This suggests that Ly6G expression positively correlates with NETs and AAA severity. SMA is a vascular smooth muscle cell marker and skeletal protein associated with cell contractility. In the normal vascular smooth muscle layer, αSMA is widely expressed in the VSMC cytoplasm. With the progression of AAA, VSMC underwent cellular phenotypic changes, with decreased αSMA expression and reduced vascular elasticity. Inhibition of NETs accumulation significantly suppressed phenotypic changes in VSMCs (Fig. 7C, F). In addition, we performed IF co-localization of Ly6G and CitH3 in the aortas of the four groups (Fig. 7G). There was a significant infiltration of neutrophils and NETs within the lesion group, which was significantly reduced in the drug-treated group.

Besides, IF staining showed that NETosis markers (MPO and CitH3) were expressed in the arterial wall of AAA lesions (Fig. S9A). EVG staining of surgically obtained AAA lesion tissue showed thinning and loss of elastic fibers in the arterial wall (Fig. S9B).

## Discussion

The most important factor causing AAA progression and rupture is inflammation of the blood vessel wall. Currently, the lack of functional imaging modalities that can accurately identify vascular inflammation is a major limitation in AAA. This study demonstrated that Ly6G NPs can be used for MPI/FLI/CT multimodal imaging targeting neutrophils to detect AAA.

The principal pathological characteristics of AAA are ECM degradation, impaired VSMC function, and, ultimately, loss of good contractility of the aorta [4]. The infiltration of immune cells accounts for these features, leading to expansive remodeling of the aortic wall, continuous loss of elasticity of the aortic wall, and the formation of blood clots within the aortic lumen [30, 31]. Thus, the progression of AAA and the change in aortic wall strength are significantly influenced by inflammatory processes. The neutrophil is the most plentiful circulating leukocyte in the human immune system and serves as the primary barrier against internal immunity; its main functions include phagocytosis, degranulation, and release of NETs [32]. When stimulated, activated neutrophils respond rapidly, within tens of minutes, by undergoing NETosis and releasing proteins with multiple functions containing neutrophil elastase (NE), MPO, histone G, IL37, and gelatinase [33]. NETs are critical for AAA development. (1) NETs are enriched with matrix metalloproteinases (MMPs) that degrade collagen fibers and elastic fibers in the extracellular matrix and disrupt intercellular junctions [34]. (2) NETs promote the conversion of VSMC to an anabolic and pro-inflammatory phenotype by suppressing the Hippo-YAP pathway, which not only diminishes the elasticity of the tubular wall but also aggravates inflammation [27]. (3) NETs enhance the transcription of IL-6 and IL-1β in macrophages, activate plasma cell-like dendritic cells, trigger the differentiation of Th17 cells, and draw in additional inflammatory cells [25, 35]. (4) NETs are cytotoxic and will directly kill vascular endothelial cells (ECs) and VSMCs [36]. (5) In addition, NETs can trap blood cells and accumulate on the vessel wall, ultimately leading to intraluminal thrombosis and promoting vascular occlusion [37]. In a mouse AAA model, the inhibition of NETosis by degradation of NETs using DNase I or blocking PADI4 with YW3-56 and Cl-Amidine significantly attenuated AAA formation in vivo [38, 39]. These studies demonstrated that neutrophils are involved in the early onset and progressive deterioration of AAA and are important targets that may be used for functional imaging of AAA in the future.

Morphologic imaging of vascular diameter remains the primary reference standard for the detection and management of AAA, typically defined as the infrarenal abdominal aorta having the largest diameter of 30 mm or greater on ultrasound or CT imaging [40]. Current guidelines recommend conservative management of small asymptomatic AAA (less than 55 mm in diameter for males and less than 50 mm in diameter for females) with regular monitoring of AAA diameter and patient symptoms [6]. If the aneurysm manifests symptoms or expands to a maximum diameter of 55 mm, surgery, and endoluminal repair should be considered. With the development of imaging techniques in the last decade, many methods for identifying AAA with high rupture risk have been explored. Since the breakdown of proteins in the aortic wall due to chronic inflammation is considered a primary factor in the reduction of aortic wall elasticity and the expansion of aneurysms, inflammation has become an important area of functional imaging studies of AAA [41]. Gregory et al. injected AAA mice with the USPIO contrast agent Ferumoxtran-10 for in vivo MRI and found that the signal was altered after the phagocytosis of USPIO by macrophages within the aneurysm, demonstrating that the USPIO contrast agent can serve as a tool for accessing acute inflammatory events associated with the progression of AAA [42]. Maarten et al. assessed the presence of USPIO in the MRI images of six patients with aortic aneurysms and five patients with iliac aneurysms and found that only the aortic aneurysm wall with extensive macrophage infiltration exhibited high levels of USPIO uptake [43]. In a forward-looking, multi-site cohort investigation, 342 individuals with AAA (≥40 mm in diameter) were classified according to USPIO enhancement and then subjected to repeated ultrasound surveillance and continued clinical evaluations for a minimum of 2 years [44]. These findings imply that MRI enhanced with USPIO represents an innovative technique for identifying inflammation within the aortic wall of patients with AAA and forecasting the rate of aneurysm expansion and the potential clinical outcomes. In addition, MRI can identify AAA intraluminal thrombi and angiogenic uptake of USPIO [45, 46]. These studies have greatly contributed to the development of functional imaging of AAA, which offers a promising new strategy for the assessment and classification of patients with AAA that goes beyond the straightforward measurement of the aneurysm’s diameter.

Previous functional imaging based on inflammation detection focused on MRI, demonstrating the uptake of USPIO by the inflammatory sites of AAA lesions [15, 41]. The main drawback of this approach is that USPIO results in reduced signal intensity on MRI, which can easily be missed or mistaken for artifacts [47]. In contrast, MPI allows modified USPIO to detect inflammation with a higher specificity and sensitivity. There is only one study on the use of MPI technology for AAA. In their study, MRI (in vivo), MPI (ex vivo), and magnetic particle spectroscopy (MPS) (ex vivo) imaging were represented in AAA mice injected with ferucarbotran, and uptake of USPIO by macrophages within the aneurysms was confirmed by histologic analysis. Unfortunately, they did not perform in vivo MPI imaging, and USPIO was not targeted [15].

This research successfully established a mouse model by incubating the abdominal aorta of mice using elastase. With the prolongation of modeling time, AAA duct dilatation became increasingly obvious, and the expression of neutrophils and NETs in lesion tissues and peripheral circulation showed an upward trend. Therefore, we concluded that infiltrating neutrophils and NETs could be used as markers of AAA progression. However, noninvasive targeted detection of NETs is currently a challenge. Because both neutrophils and NETs infiltrate AAA lesions, based on previous studies, we chose Ly6G as the target of this probe [48]. Ly6G NPs consisted of anti-Ly6G, an antibody that actively targets Ly6G, iron tetraoxide nanoparticles (Fe_3_O_4_@PEG–COOH), and fluorescent moieties (Cy7–NHS). Ly6G NPs were not significantly toxic to neutrophils, as determined using CCK-8. After the tail vein of normal mice was injected with Ly6G NPs (7.5 mg Fe/kg), no significant damage was observed by pathological examination of the aorta, heart, liver, spleen, or other organs 24 h and 15 d after injection. By intramuscular injection of a Ly6G protein-containing matrix gel into the thigh muscles of mice, MPI scans revealed that Ly6G NPs were enriched in the left thigh and reached the strongest signal at 24 h. Therefore, 24 h after probe injection was used as the time of MPI scanning in AAA mice. USPIO tends to be found in the liver and spleen [16]. Using Prussian blue staining on the major organs revealed the presence of Ly6G accumulation in the spleen and liver. Therefore, the spleen and liver were resected in advance to obtain MPI imaging of Ly6G in AAA. Interestingly, ex vivo FLI showed higher fluorescence signals in the intestine, stomach, liver, spleen, and kidney, which may result from the presence of autofluorescence in the feed and drinking water of mice. In addition, due to the small size of Cy7–NHS, it then accumulates in the kidney, intestine, and stomach.

Multimodal molecular imaging is a valuable method for non-invasive observation of AAA components, disease progression, and complications in living organisms. In this study, MPI/FLI/CT, based on a multimodal probe for Ly6G NPs, was used to target the infiltrating neutrophils in AAA to reflect disease progression. Although autofluorescence can interfere with FLI and the depth of tissue penetration is limited, it has high sensitivity. FLI has been used to image atherosclerotic plaques with the superficial arteries, including those in the abdominal aorta and carotid arteries [16, 49]. MPI can overcome these drawbacks because it is sensitive, independent of the tissue depth, and does not ionizing radiation [50]. However, neither is capable of morphologic imaging of tissue structures and can detect the diameter of the aortic lumen, provide anatomical structures, or assist in achieving 3D fusion imaging. Thus, MPI/CT not only enables imaging of the entire aorta but also allows sensitive identification of inflammation in AAA lesions with 3D whole-body imaging. As the diameter of the abdominal aorta in humans is considerably wider than that in mice, future clinical imaging may be better. In addition, light-sheet fluorescence microscopy for 3D fluorescence imaging of the entire aorta allows for a clearer view of the composition of components within the AAA and the localization of the probe distribution. In addition, it can be used to monitor improvements in AAA after inhibiting the release of NETs. This demonstrates that MPI has enormous potential for detecting inflammation at AAA lesion sites and monitoring the expression of NETs.

Our study has several limitations. First, imaging AAA lesions using MPI requires resection of the liver and spleen. Similar to the distribution patterns of other Fe_3_O_4_-based contrast agents, Ly6G nanoparticles were mainly located in the liver and spleen following histological examination [16]. Owing to the small size of the mouse, the aorta is very close to the liver, which makes MPI signaling in iron-accumulating organs problematic. However, in the human body, the liver and spleen are distant from the site of the AAA lesion, and it may be possible to improve the quality of the imaging by altering the scanning area. Therefore. We are confident that MPI can be used to detect AAA in future clinical trials. In addition, the design of Ly6G NPs was improved to enhance their metabolism in the liver and promote the stock of the probes in AAA lesions to reinforce their sensitivity and specificity. Second, MPI is still a preclinical imaging method that needs to be used in conjunction with CT to provide anatomical data for the precise localization of the imaged area. This requires transferring the mice from the MPI scanner to the CT scanner, which can lead to spatial matching errors and requires image fusion processing. Finally, the MPI/FLI/CT imaging of Ly6G NPs in the current study was only relevant to mouse models. As SPIONs become increasingly utilized for MPI and MRI imaging, ferumoxytol and ferucarbotran have also been approved for use in clinical research. For humans, further optimization of the design of probes targeting NETs is required to confirm their biosafety and efficacy.

Overall, we successfully designed specific Fe_3_O_4_ nanoprobes (Ly6G NPs) that are available for MPI/FLI/CT multimodal imaging of neutrophils in AAA. In addition, Ly6G NPs could be used to identify the extent of AAA lesions and monitor neutrophil infiltration.

## Material and methods

### Synthesis of PEG–Fe_3_O_4_–Ly6G–Cy7

First, 10 mg of 20 nm Fe_3_O_4_ (1 mg/mL) was centrifuged in a centrifuge tube (14,000*g*, 30 min), and the supernatant was discarded, washed once with MES, and resuspended in 9.49 mL. Then 100 μL of EDC (10 mg/mL) was vortexed into Fe_3_O_4_, and 1 mg of Ly6G antibody (Biolegend, 127632) (2.43 mg/mL, 410 μL) was added after mixing, and the reaction was shaken at 37 °C for 4 h. When the reaction finished, the reaction solution was separated, and the supernatant was obtained through centrifugation (for detection of the antibody coupling rate). The supernatant was removed by centrifugation, then the magnetic material was washed three times using purified water and then fixed to 5 mL, which yielded magnetic nanoparticles, denoted as 20 nm PEG–Fe_3_O_4_–Ly6G. Add 0.5 mg Cy7–NHS and shake the bed at 37 °C for 1 h, then centrifuged and discarded the supernatant. The magnetic material was washed with purified water three times and then fixed to 10 mL to obtain magnetic nanoparticles, denoted as 20 nm PEG–Fe_3_O_4_–Ly6G–Cy7 (Ly6G NPs). Synthetic materials were provided by Nanjing NanoEast Biotech Co. Besides, PEG–Fe_3_O_4_–BSA–Cy7 (BSA NPs) was constructed with bovine serum albumin (BSA) (Yeasen, 36101ES76) instead of Ly6G as a negative control.

### Characterization of PEG–Fe_3_O_4_–Ly6G–Cy7

Core dimensions of Ly6G NPs were measured using transmission electron microscopy (TEM) (JEM-1200EX, JEOL, Tokyo, Japan). The hydrodynamic dimensions and zeta potential values of Ly6G NPs were then examined at room temperature. The fluorescence emission spectra of Ly6G NPs were recorded at 700 nm excitation light excitation (Hitachi, Tokyo, Japan). Then the X-ray diffraction (XRD) patterns of Ly6G NPs were analyzed using an X-ray powder diffractometer (D8 ADVANCE, BRUCKER, Germany). Finally, Ly6G NPs were dispensed into different concentrations in 96-well plates and their MPI and FLI signal values were detected, respectively.

### Animal model of abdominal aortic aneurysms

C57BL/6 J mice (male, 8–10 weeks old) were purchased from Beijing Viton Lihua Laboratory Animal Technology Co. Referring to the previous method, elastase incubation of AAA in mice was used for modeling [51]. Six mice per group were examined ultrasonographically at 1–4 weeks after modeling to observe AAA formation. Researchers were not blinded to the group of the animals. An aortic aneurysm model was considered successful if dilation of the aortic diameter exceeded 50%. To test whether Ly6G NPs could monitor the onset and development of early AAA, 3 d and 1 w mice, after modeling, were injected with probes and subjected to multimodal imaging.

Cl-Amidine hydrochloride, a peptidylarginine deiminase 4 (PADI4) inhibitor, blocks citrullination of histone 3 and the formation of NETs. Cl-Amidine (10 mg/kg) was injected intraperitoneally daily for 3 weeks during AAA modeling. Deoxyribonuclease (DNase I) is an enzyme that degrades DNA and can degrade NETs products. DNase I (4 mg/kg) was injected intraperitoneally daily for 3 weeks during AAA modeling. All drugs were purchased from MedChemExpress.

### Clinical samples of abdominal aortic aneurysms

AAA human samples were obtained from the Vascular surgery department of the Second Hospital of Shanxi Medical University, and all samples followed the application of human ethics.

### Cell culture and cytotoxicity studies

Mice peripheral blood neutrophils were extracted using the EasySep Mouse Neutrophil Isolation Kit (STEMCELL, Catalog # 19762). The cells were cultured in Peripheral Blood Neutrophil Complete Medium (CM-H197). The cytotoxicity of Ly6G NPs was evaluated by the Cell Counting Kit-8 (CCK-8) (Beyotime, C0037). Ly6G NPs were dissolved in culture medium at iron concentrations of 0, 5, 10, 25, 50, 100, 150, 200, and 250 μg/mL. Six hours later, neutrophils were rinsed three times with PBS. Then 10 μL of CCK-8 solution was added to 100 μL of PBS and incubated for 1 h. Finally, the optical density was measured at 450 nm.

### In vivo targeting validation of Ly6G NPs

A 100 µl of matrix gel containing Ly6G (10 µg) and lipopolysaccharide (5 units) was injected into the left thigh muscle of mice (*n* = 3). As a control, a mixture containing 100 µl of PBS was injected into the right thigh. Lipopolysaccharide was used to generate an inflammatory response, and matrix gel was used to immobilize Ly6G protein and lipopolysaccharide. Twelve hours after injection of the mixture, Ly6G NPs (7.5 mg Fe/kg) [16] were injected into the tail vein of mice for in vivo imaging.

### MPI imaging

MPI signals were acquired using an MPI scanner (MOMENTUM, Alameda, USA). The parameters for two-dimensional (2D) MPI imaging were as follows: field of view: 4 cm × 4 cm; scanning modes: isotropic (in vivo) and high sensitivity (ex vivo); magnetic field gradient strength: 5.7 T/m; total time: 2 min. Three-dimensional (3D) MPI imaging was performed with the following parameters: field of view: 6 × 6 × 10 cm; scan mode: isotropic; total time: 35 min. MPI images were analyzed using VivoQuant software (VivoQuant 4.0, Invicro, Boston, MA, USA).

### Fluorescence imaging

Fluorescence imaging (FLI) was performed using a small animal in vivo imaging system (IVIS) to detect Cy7 fluorescence signals in mice. The excitation and emission wavelengths were 720 nm and 780 nm, respectively. The mice were depilated because the black hair would affect the fluorescence signal. Fluorescence intensity was quantified in vivo or ex vitro using Living Image software (Caliper Life Sciences).

### CT imaging

CT imaging of AAA mice injected with Ly6G NPs was performed using a micro CT scanner (PerkinElmer, Waltham, USA). Scanning parameters were: voltage: 120 kV; current: 80 μA; field of view: 45 mm; voxel size: 1.5 mm; scanning mode: high resolution; and scanning time: 30 min. Finally, the 3D data from MPI was fused and reconstructed with CT data using VivoQuant software.

### Ultrasound imaging

To assess the diameter of the abdominal aorta in mice, it was monitored by ultrasound using the Vevo 2100 platform (Visual Sonics, Toronto, CA, USA) [52]. Data were collected and processed using a double-blind method and instrumented with normal mice.

### Tissue clearing and light-sheet microscopy imaging

Segments of mouse abdominal aorta were embedded in 1% agar and dehydrated in 25, 50, 75, 100, and 100% methanol for 3 h per step. The arteries were washed overnight with benzyl alcohol and benzyl benzoate (1:2). All steps were performed in the dark at 20 °C.

Multidimensional fluorescence image acquisition using light-sheet microscopy (LSM) (Ultramicroscope II, LaVision Biotec, Bielefeld, Germany). Abdominal aortic tissue was placed on a plastic holder and submerged in a glass dish containing an imaging solution configured with benzyl alcohol and benzyl benzoate (1:2). The signal channels for each fluorescence are respectively: Alexa Fluor® 700 anti-mouse Ly6G Antibody (BioLegend, 127622): Ex: 633 nm, Em: 719 nm; FITC Anti-Myeloperoxidase antibody (abcam, ab90812): Ex: 493 nm, Em: 528 nm; APC anti-mouse CD34 Antibody (BioLegend, 128612): Ex: 651 nm, Em: 660 nm; Alexa Fluor 488 anti-mouse αSMA Antibody (Invitrogen, Cat#53-9760-82): Ex: 499 nm, Em: 520 nm. Finally, 3D reconstruction was performed using Image J software.

### Histopathological staining

Hematoxylin and eosin and Prussian blue staining. After ex vivo FLI and MPI ex vivo, the abdominal aorta and major organs (liver, spleen, kidney, stomach, colon) were harvested and then fixed with 4% paraformaldehyde and paraffin-embedded for histological sections (5 μm). The sections were stained with hematoxylin and eosin (H&E), Masson’s trichrome reagent, and Prussian blue according to standard routine protocols.

EVG staining of elastic fibers of the abdominal aorta was performed to assess the degree of elastic fiber fracture. Briefly, arterial sections were deparaffinized in water, stained with Verhöeff’s staining solution at 37 °C for 15 min, rinsed with running water, and differentiated with Verhöeff’s differentiation solution for 10–20 s until the elastic fibers were clear (observed under the microscope). The sections were then rinsed thoroughly under running water, restrained in Van Gieson’s solution for 30 s, dehydrated in anhydrous ethanol, made transparent in xylene, and fixed in a neutral resin.

### Immunofluorescence staining

Immunofluorescence (IF) staining was performed on paraffin sections of mouse abdominal aortic tissue. Co-localization of the two target proteins was performed as follows: dewaxing, antigen repair and serum blocking. The first primary antibody was then added and incubated overnight at 4 °C in a wet box, followed by fluorescent labeling with Cy3. The second primary antibody was then added and incubated overnight at 4 °C in a wet box, followed by fluorescent labeling with FITC. The nuclei were then stained with DAPI. Finally, image acquisition is performed. If there is only one primary antibody, reduce the corresponding step. The following antibodies were used: anti-Ly6G (Servicebio, Wuhan, China), anti-CitH3 (Abcam, London, UK), and anti-MPO (Proteintech, Wuhan, China).

### Biochemical analyses

The test kits for aspartate aminotransferase (AST/GOT) (C010-2-1), alanine aminotransferase (ALT/GPT) (C009-2-1), Creatinine (C011-2-1) and BUN (C013-2-1) were supplied by the Nanjing Jiancheng Bioengineering Institute (Nanjing, China). All operating procedures are carried out with strict reference to the standards of the manual.

### Western blot

Total cellular protein was extracted using an enhanced RIPA lysis buffer. Proteins were separated using a 10% SDS-PAGE gel and then transferred to a polyvinylidene difluoride (PVDF) membrane. Protein expression was detected using primary antibodies against ELANE (Boster Biotech, Wuhan, China), MPO (Proteintech, Wuhan, China) and GAPDH (Servicebio, Wuhan, China). The primary antibody was left to incubate overnight at 4 °C, while the secondary antibody was incubated at room temperature for 1.5 h. It was then rinsed with ECL reagent for 30 s and analyzed using a gel imaging system (Bio-Rad, California, USA).

### Statistical analysis

The statistical analysis was performed using SPSS software. Data were expressed as mean ± standard deviation. If the experimental data were normally distributed, a *t*-test was used for comparison between two groups, and a one-way analysis of variance (ANOVA) was used for comparison between multiple groups. If the experimental data were non-normally distributed, the Mann–Whitney *U* test was used. Analyses between each experimental group were performed using Tukey’s test or Dunn’s multiple comparison test. A *p*-value less than 0.05 was considered statistically significant.

## Supplementary information

Video 1

Video 2

Supplemental Material

Original Western Blots

## Data Availability

The original contributions presented in the study are included in the article. Further inquiries can be directed to the corresponding authors.
