# Supplementary material for: Highly sensitive magnetic particle imaging of abdominal aortic aneurysm NETosis with anti-Ly6G iron oxide nanoparticles
Source: Cell Death Discov. 2024 Sep 5;10:395. doi: 10.1038/s41420-024-02156-3 (PMC11377588; doi:10.1038/s41420-024-02156-3)
Supplement: Supplementary file 3 — Supplemental Material [file 41420_2024_2156_MOESM3_ESM.pdf]

Supplementary Figures

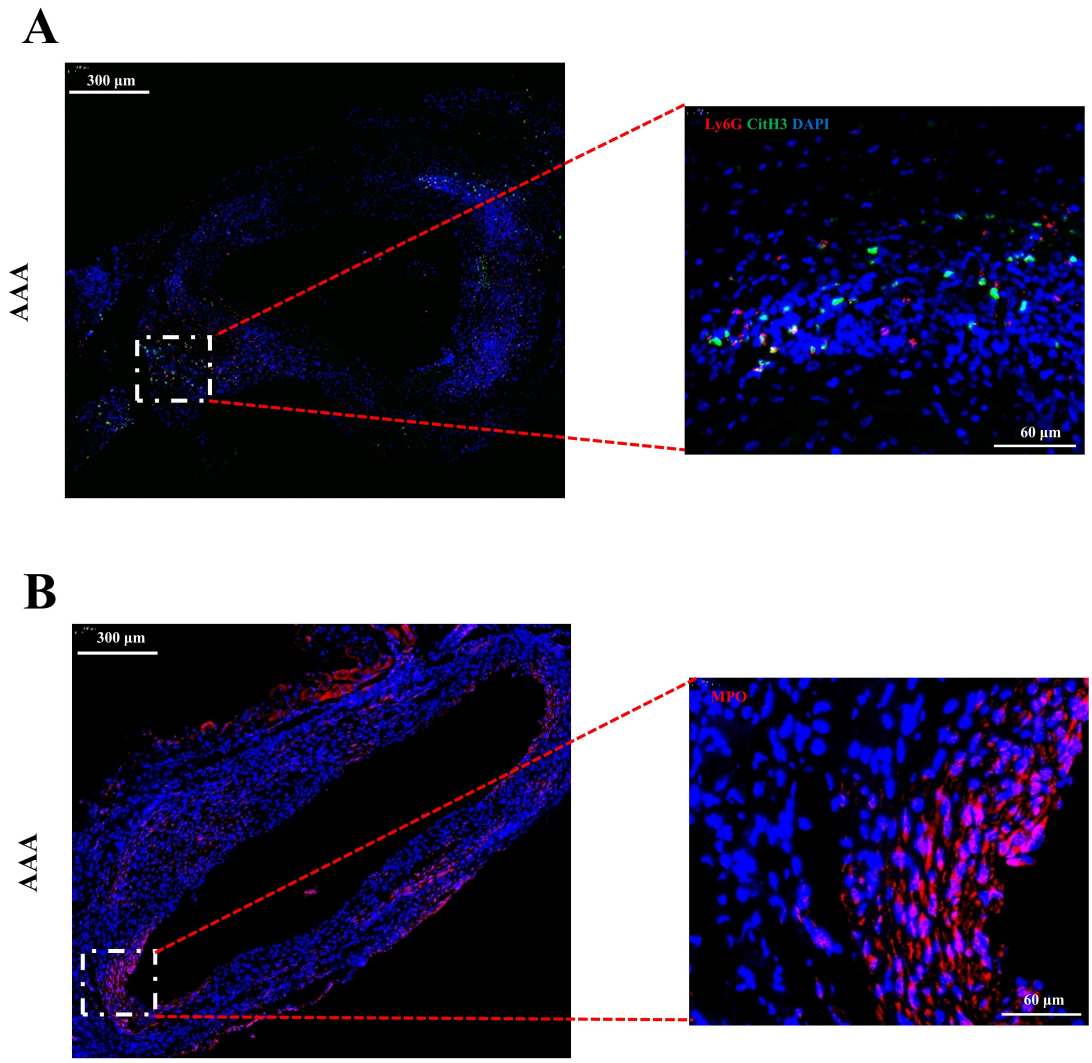

Fig. S1 The co-localization of neutrophils and NETs markers at lesion sites in AAA mice. (A) IF co-localization staining for neutrophil marker (Ly6G) and NETs marker (CitH3) was performed on AAA tissue sections. **(B)** IF staining for NETs marker (MPO) on AAA tissue sections.

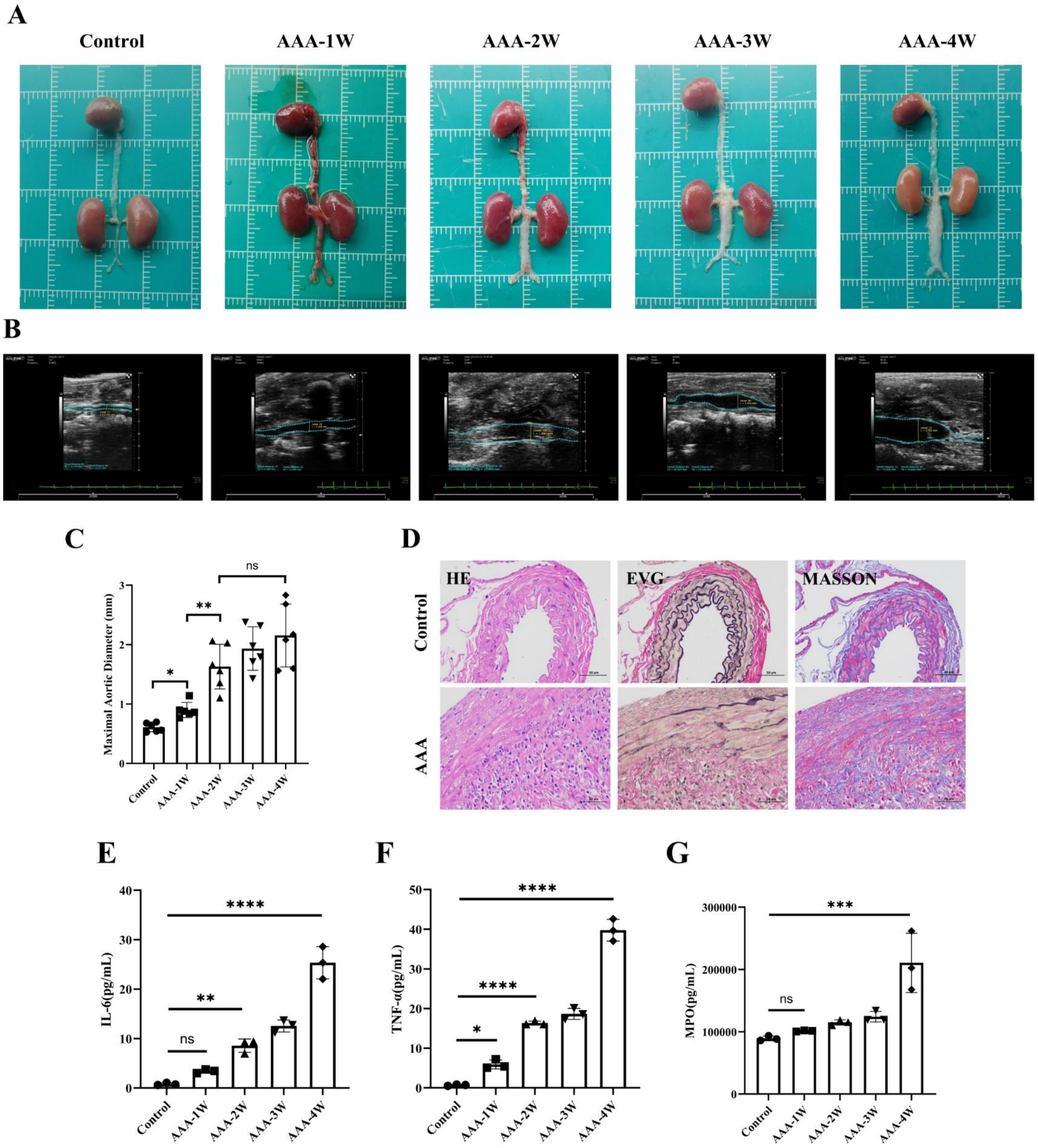

Fig. S2 The AAA model was successfully established. General photos **(A)** and vascular ultrasound images **(B)** of abdominal aorta at different modeling time. **(C)** According to the ultrasonic data of blood vessels, the internal diameter of blood vessels is quantitatively analyzed (n=6). **(D)** Pathological staining of paraffin sections of abdominal aorta (HE, EVG and MASSON). **(E-G)** Serum inflammatory indexes (IL-6 and TNF-α) and NETs markers (MPO) were detected by ELISA (n=3). *P < 0.05, **P < 0.01, ***P < 0.001, ****P < 0.0001, indicating statistically significant data between groups; ns indicates no statistical significance.

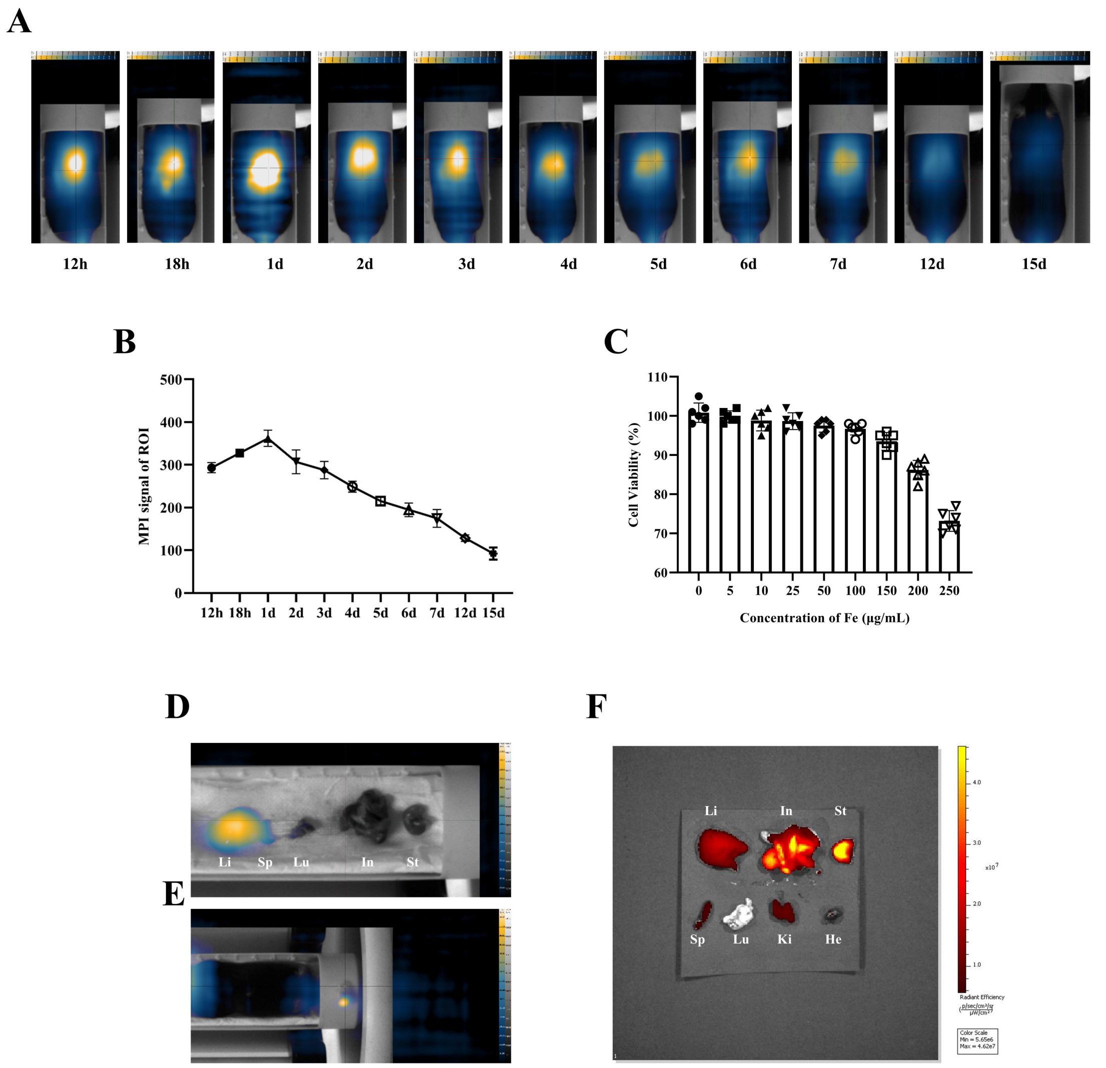

Fig. S3 The *in vivo* metabolism and cellular safety of Ly6G NPs. (A, B) Metabolism of Ly6G NPs (7.5 mg Fe/kg) injected into normal mice via tail vein from 12 h to 15 d (n=3). **(C)** Cytotoxicity of human peripheral blood neutrophils in Petri dishes after incubation with different concentrations of Ly6G NPs (n=6). **(D)** Viscera were obtained for MPI imaging after Ly6G NPs were injected for 24 h and mice were executed. **(E)** At 24 h of Ly6G NPs injection, MPI imaging was performed on mice with liver and spleen removed. Li: liver; Sp: spleen; Lu: lung; In: intestine; St: stomach; Ki: kidney; He: heart. **(F)** FLI imaging of each organ in mice at 24 h of Ly6G NPs injection.

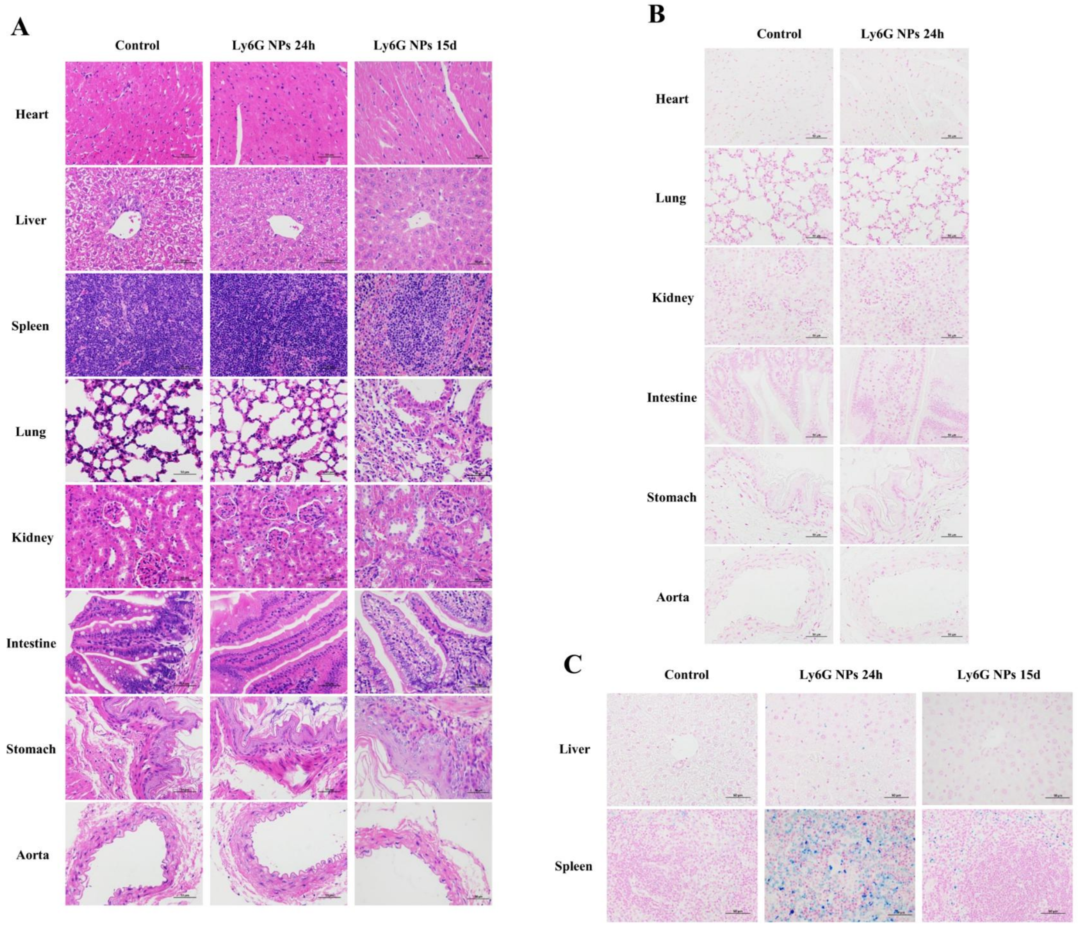

Fig. S4 Pathological staining of various organs in mice after injection of Ly6G NPs. (A) Ly6G NPs (7.5mg Fe/kg) was injected into normal mice by tail vein, and paraffin sections of various organs were stained with HE. **(B, C)** Ly6G NPs was injected into normal mice via tail vein, and the paraffin sections of various organs were stained with Prussian blue.

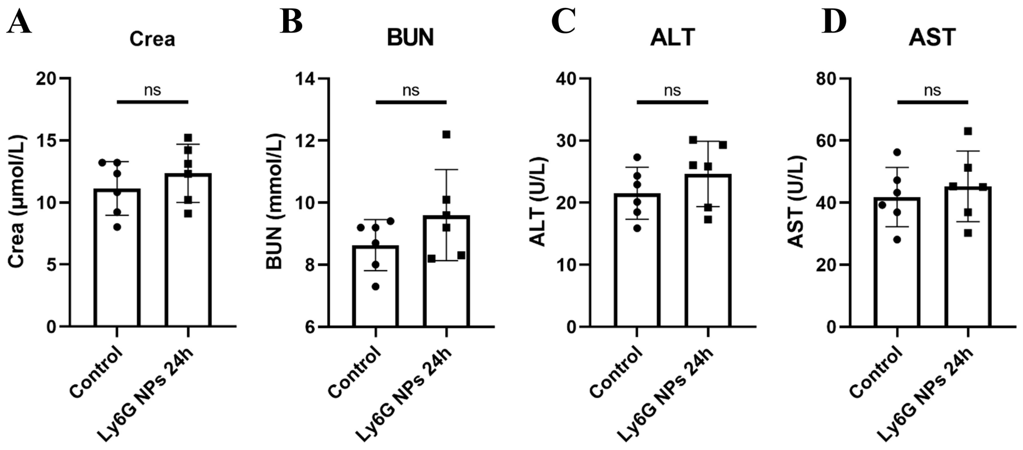

Fig. S5 Hepatic and renal function tests demonstrate the biocompatibility of Ly6G NPs. (A-B) Hepatic function tests including serum creatinine (Crea) and blood urea nitrogen (BUN) levels were detected (n=6). **(C-D)** Renal function tests including alanine aminotransferase (ALT), and aspartate aminotransferase (AST) were detected (n=6).

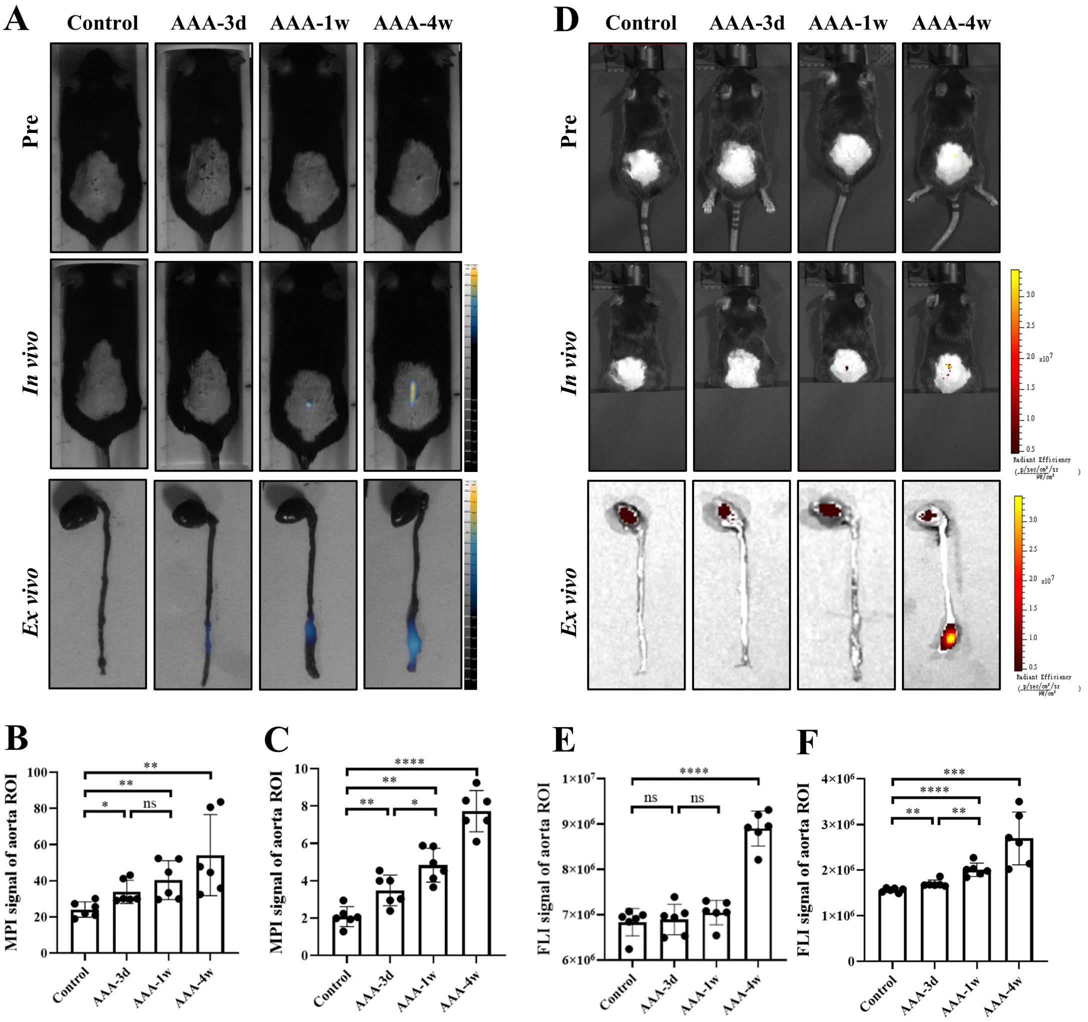

Fig. S6 MPI/FLI signals of AAA-3d, AAA-1w, AAA-4w, and healthy control mice at 24 hours after injection of Ly6G NPs. (A-C) MPI *in vivo* and *ex vivo* imaging at 24 h after injection of Ly6G NPs (n=6). **(D-E)** FLI *in vivo* and *ex vivo* imaging at 24 h after injection of Ly6G NPs (n=6). *P<0.05, **P < 0.01, ***P < 0.001, ****P < 0.0001, indicating statistically significant data between groups.

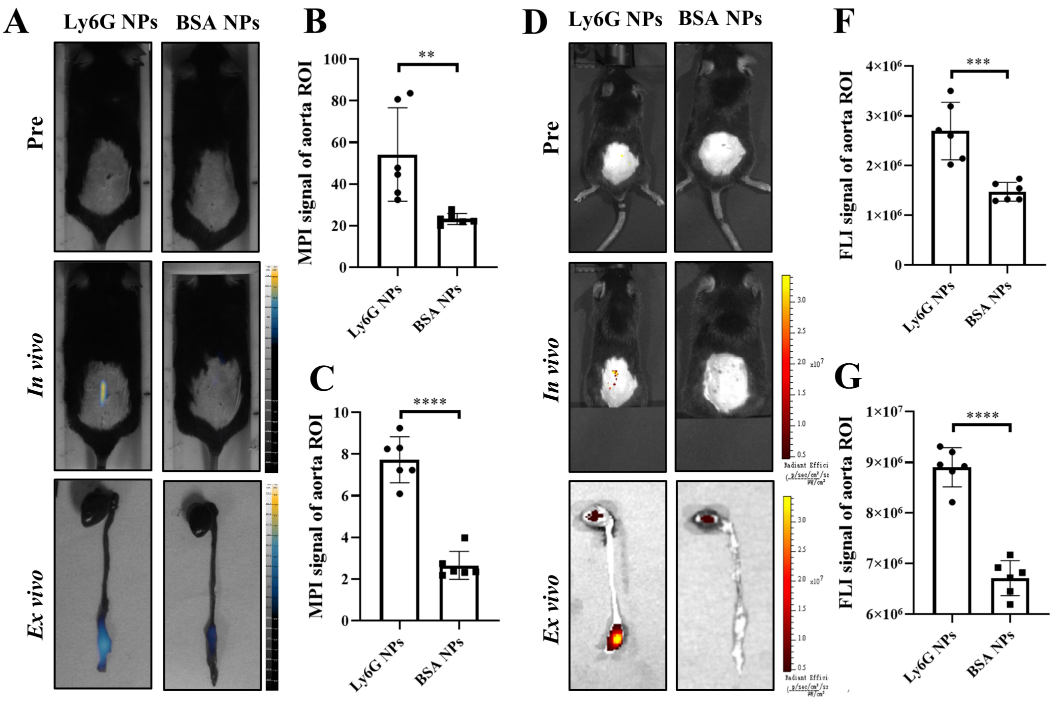

Fig. S7 MPI/FLI signals of AAA 4-week mice at 24 hours after injection of Ly6G NPs and BSA NPs. (A-C) MPI *in vivo* and *ex vivo* imaging of AAA-4w mice at 24 h after injection of Ly6G NPs (n=6). **(D-E)** FLI *in vivo* and *ex vivo* imaging of AAA-4w mice at 24 h after injection of Ly6G NPs (n=6). *P<0.05, **P < 0.01, ***P < 0.001, ****P < 0.0001, indicating statistically significant data between groups.

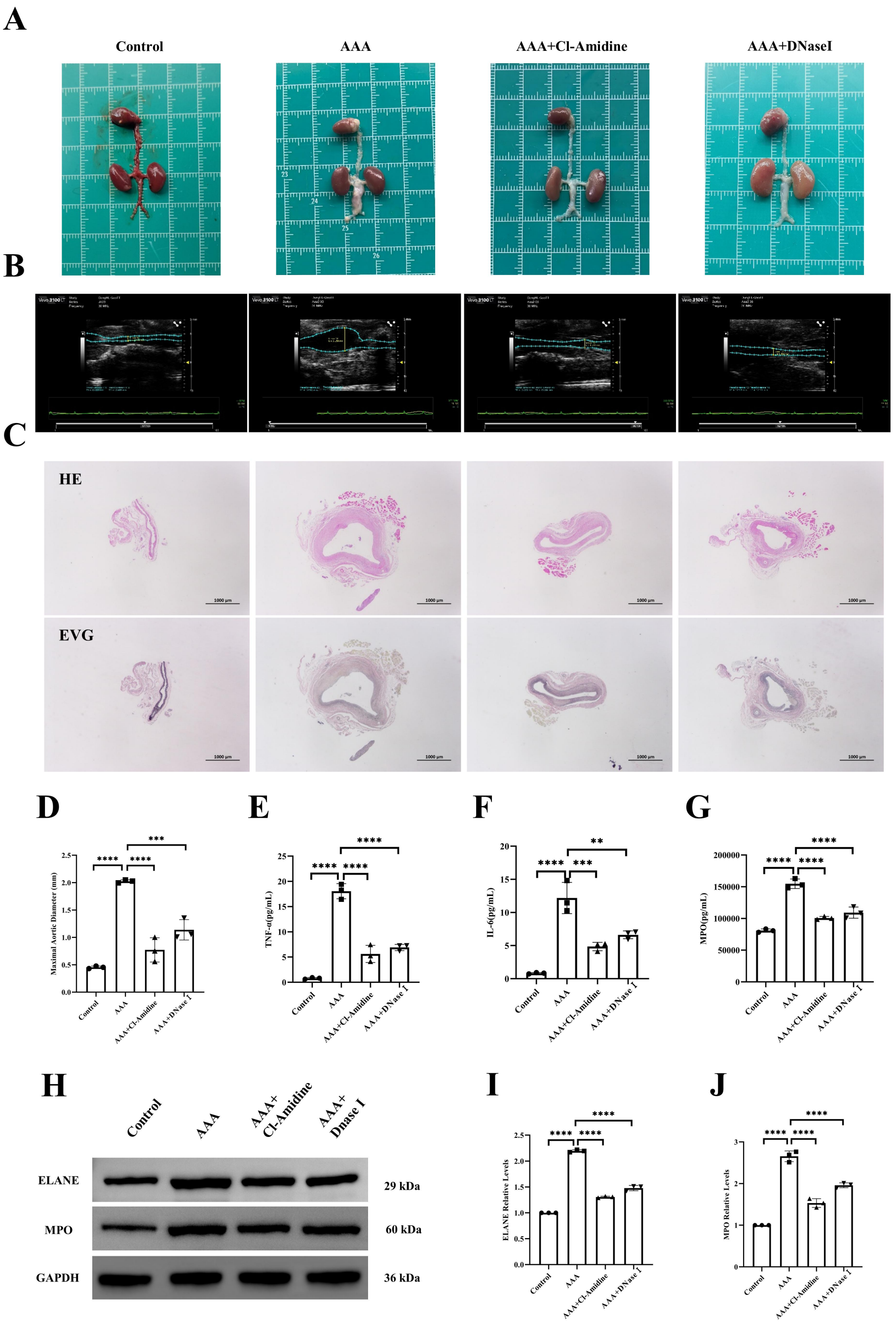

Fig. S8 Alleviate AAA by inhibiting the generation or accumulation of NETs. Gross photos **(A)** and vascular ultrasound images **(B)** of abdominal aorta in different groups of mice. **(C)** HE staining of paraffin sections of abdominal aorta of mice in each group. **(D)** According to the ultrasonic data of blood vessels, the internal diameter of blood vessels is quantitatively analyzed (n=3). **(E-G)** Serum inflammatory indexes (IL-6 and TNF-α) and NETs markers (MPO) were detected by ELISA (n=3). **(H-J)** The whole aorta was detected by western blot, and the expression of NETs products (ELANE, MPO) was quantitatively analyzed (n=3). $^{**}P < 0.01$, $^{***}P < 0.001$, $^{****}P < 0.0001$, indicating statistically significant data between groups.

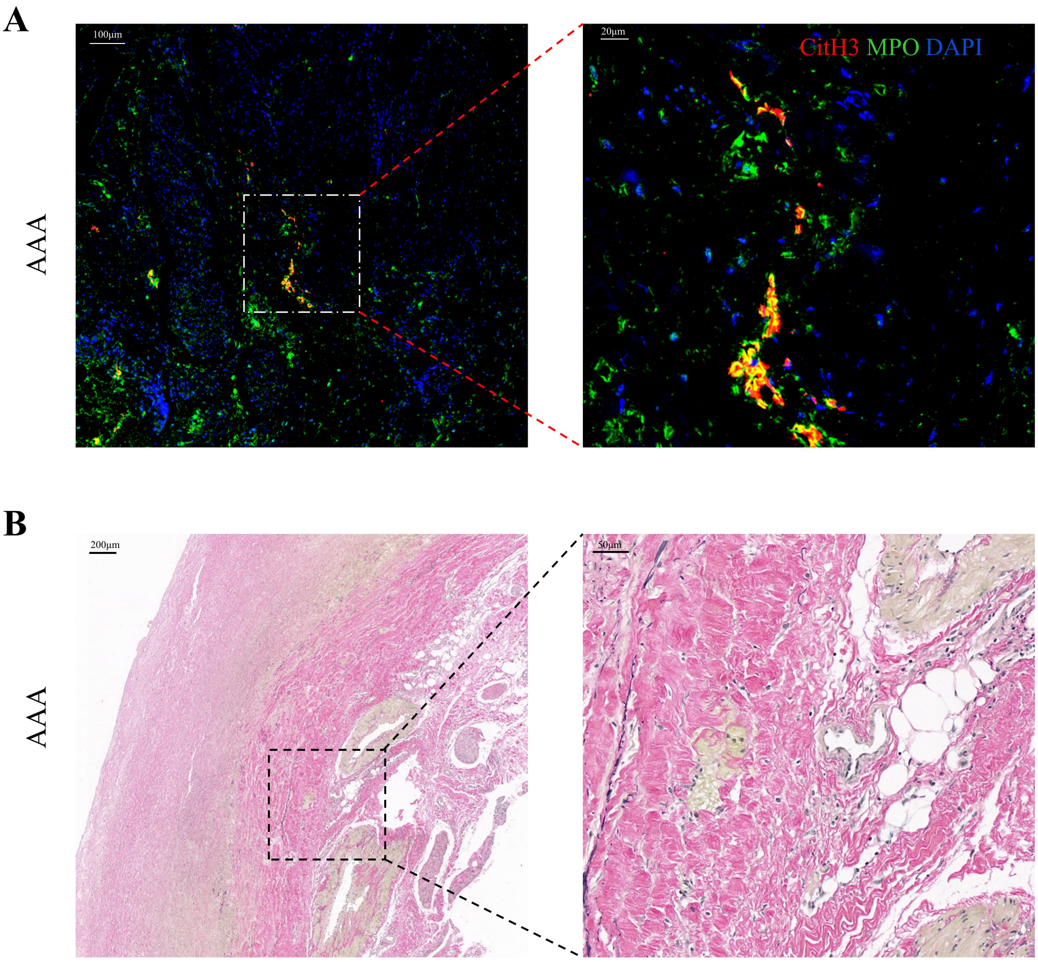

Fig. S9 Clinical samples demonstrated neutrophil infiltration and NETosis in AAA. (A) IF staining of CitH3 and MPO on abdominal aorta sections. **(B)** EVG staining of abdominal aorta sections from AAA patient. The dashed boxes are the corresponding magnified images.

Supplementary Tables

Table S1. The hydrated particle size of nanoparticles.

Sample	Name	Mean	Maximum	Minimum
**Fe_3O_4**	Intensity (nm)	100	103.6	94.42
	Number (nm)	35.71	44.98	24.04
**Ly6G NPs**	Intensity (nm)	197.6	202.1	189
	Number (nm)	105.8	137.6	84.8

Table S2. The value of zeta potential of nanoparticles.

Sample	Name	Mean	Maximum	Minimum
**Fe_3O_4**	Zeta (mV)	-27.66	-27.05	-28.02
**Ly6G NPs**	Zeta (mV)	-31.24	-30.39	-31.93

Table S3. The antibody coupling rate of Ly6G NPs.

Group	A562nm			Average	Coupling rate
**Control**	0.094	0.093	0.096	0.094	/
**Anti-Ly6G**	0.208	0.203	0.208	0.206	/
**Ly6G NPs supernate**	0.101	0.099	0.103	0.101	93.75%
