# Supplementary figures and images for: Highly sensitive magnetic particle imaging of abdominal aortic aneurysm NETosis with anti-Ly6G iron oxide nanoparticles
Source: Cell Death Discov. 2024 Sep 5;10:395. doi: 10.1038/s41420-024-02156-3 (PMC11377588; doi:10.1038/s41420-024-02156-3)

**GAPDH - Fig. 2C**


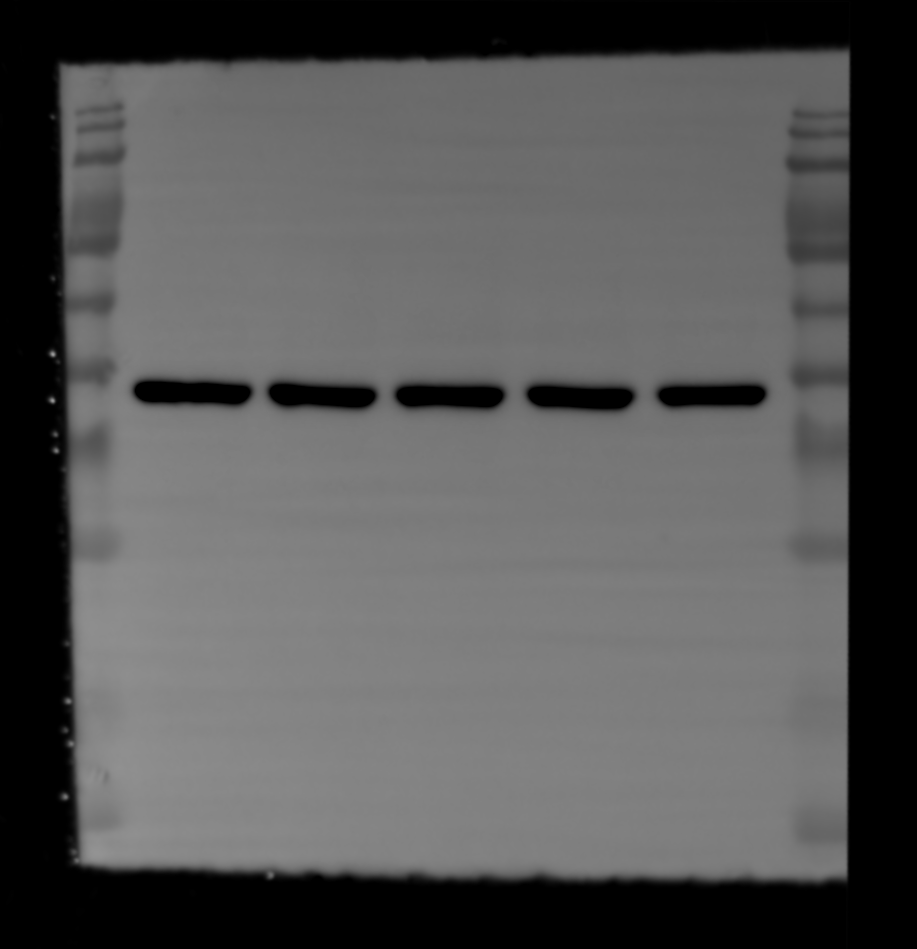


**MPO - Fig. 2C**


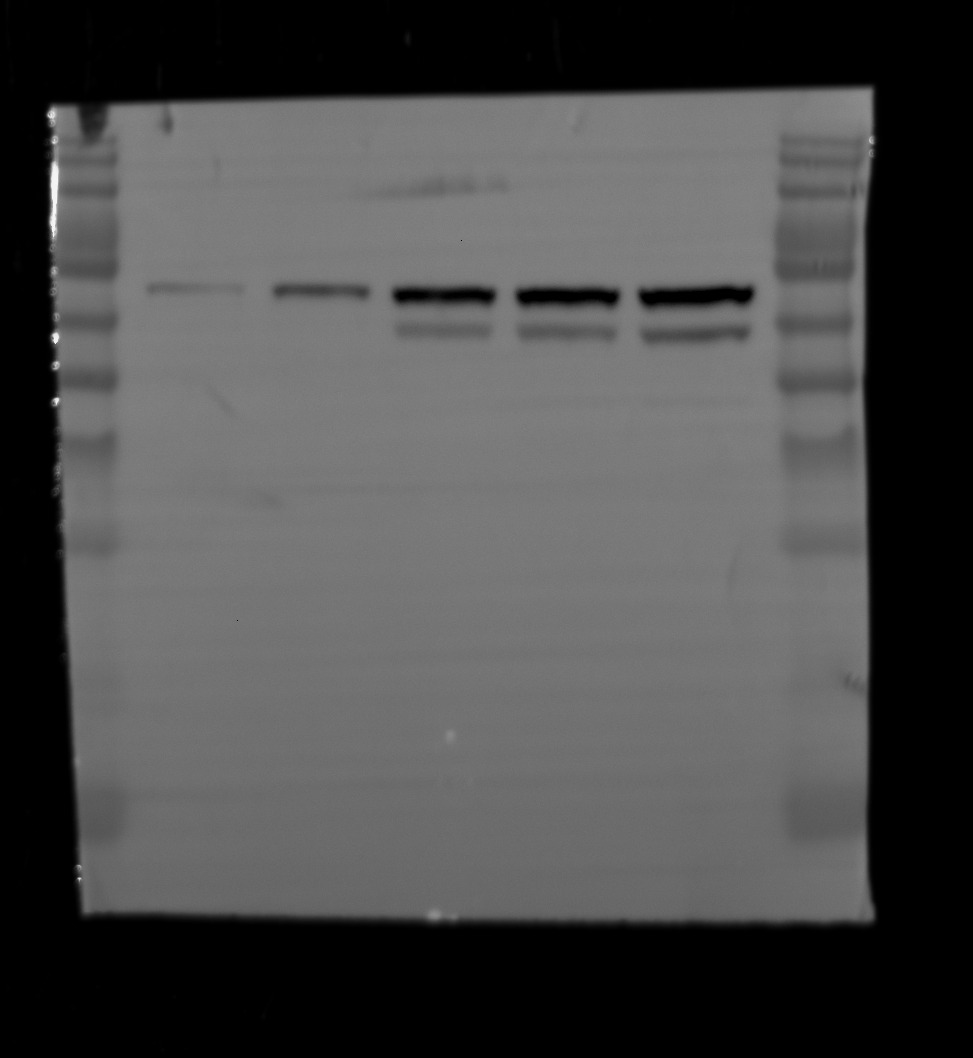


**ELANE - Fig. 2C**


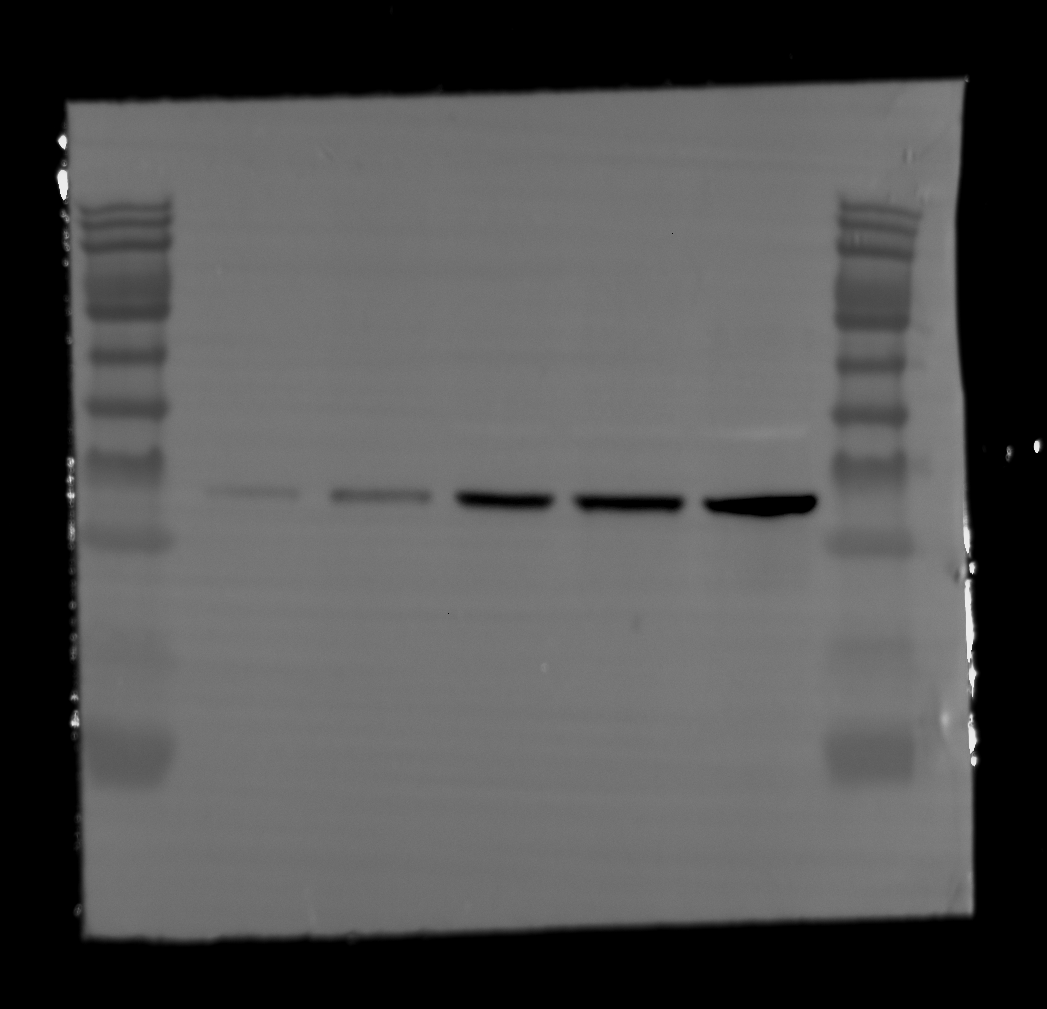


**GAPDH - Fig. S8H**


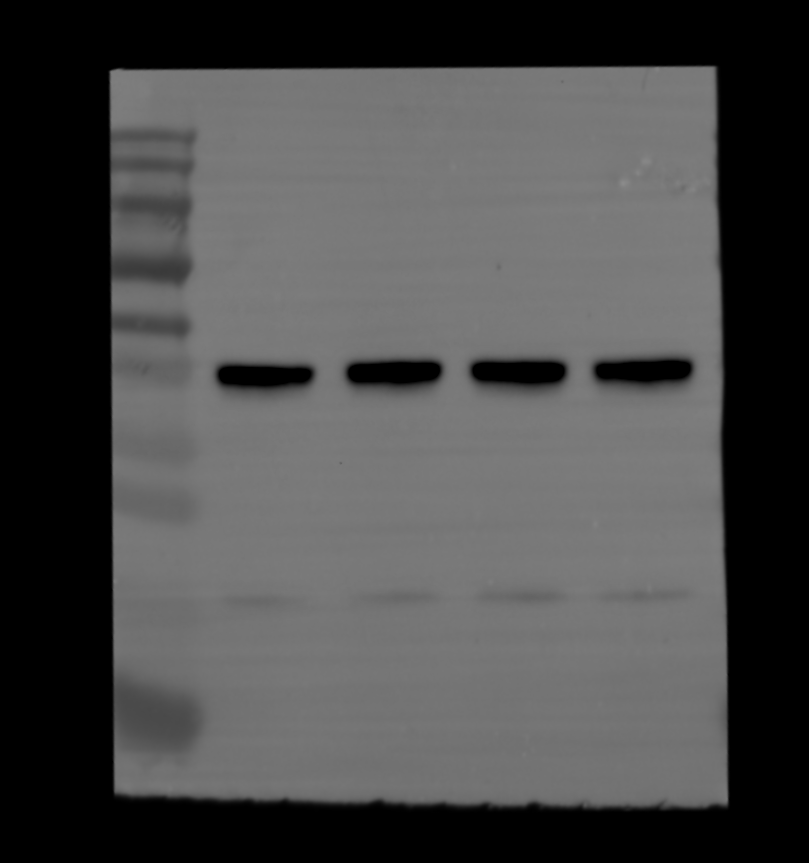


**MPO - Fig. S8H**


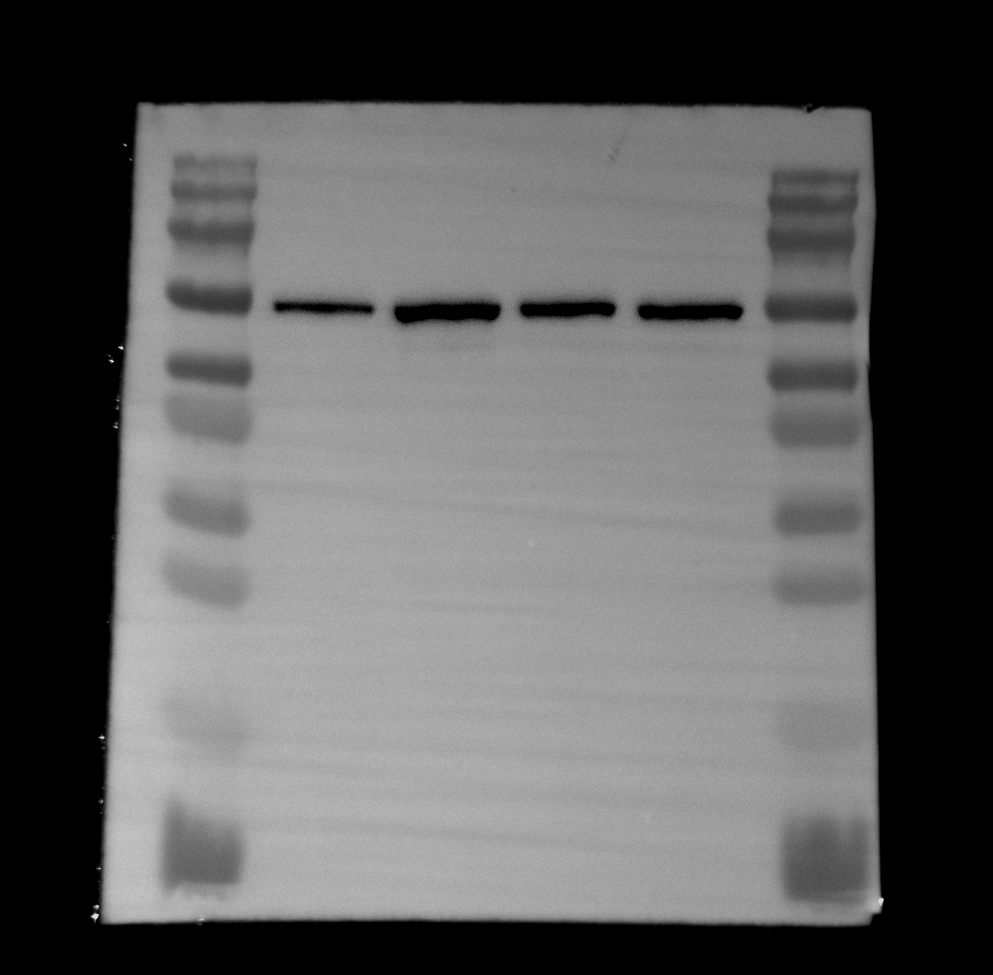


**ELANE - Fig. S8H**


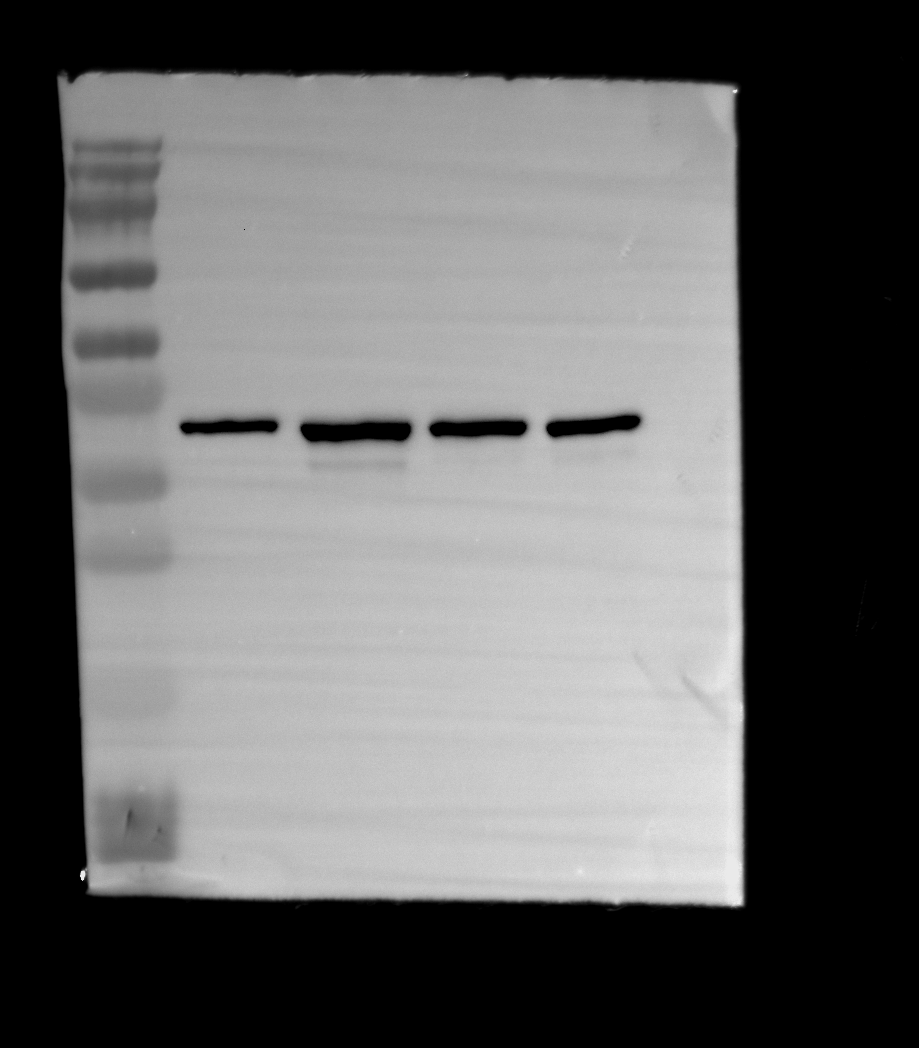

Supplement: Supplementary file 4 — Original Western Blots [file 41420_2024_2156_MOESM4_ESM.docx]
